# Sorting nexin 10 sustains PDGF receptor signaling in glioblastoma stem cells via endosomal protein sorting

**DOI:** 10.1172/jci.insight.158077

**Published:** 2023-03-22

**Authors:** Ryan C. Gimple, Guoxin Zhang, Shuai Wang, Tengfei Huang, Jina Lee, Suchet Taori, Deguan Lv, Deobrat Dixit, Matthew E. Halbert, Andrew R. Morton, Reilly L. Kidwell, Zhen Dong, Briana C. Prager, Leo J.Y. Kim, Zhixin Qiu, Linjie Zhao, Qi Xie, Qiulian Wu, Sameer Agnihotri, Jeremy N. Rich

**Affiliations:** 1 Division of Regenerative Medicine, Department of Medicine, UCSD, La Jolla, California, USA.; 2 Department of Pathology, Case Western Reserve University School of Medicine, Cleveland, Ohio, USA.; 3 UPMC Hillman Cancer Center, Pittsburgh, Pennsylvania, USA.; 4 Sanford Burnham Prebys Medical Discovery Institute, La Jolla, California, USA.; 5 Department of Neurosurgery, University of Pittsburgh Medical Center, Pittsburgh, Pennsylvania, USA.; 6 John G. Rangos Sr. Research Center, Children’s Hospital of Pittsburgh, Pittsburgh, Pennsylvania, USA.; 7 La Jolla Institute for Immunology, La Jolla, California, USA.; 8 Key Laboratory of Growth Regulation and Translational Research of Zhejiang Province, School of Life Sciences, Westlake University, Hangzhou, Zhejiang, China.; 9 Westlake Laboratory of Life Sciences and Biomedicine, Hangzhou, Zhejiang, China.; 10 Institute of Basic Medical Sciences, Westlake Institute for Advanced Study, Hangzhou, Zhejiang, China.; 11 Sanford Consortium for Regenerative Medicine, La Jolla, California, USA.; 12 Department of Neurology, University of Pittsburgh, Pittsburgh, Pennsylvania, USA.; 13 Department of Neurosciences, UCSD, La Jolla, California, USA.

**Keywords:** Oncology, Stem cells, Brain cancer, Epigenetics, Growth factors

## Abstract

Glioblastoma is the most malignant primary brain tumor, the prognosis of which remains dismal even with aggressive surgical, medical, and radiation therapies. Glioblastoma stem cells (GSCs) promote therapeutic resistance and cellular heterogeneity due to their self-renewal properties and capacity for plasticity. To understand the molecular processes essential for maintaining GSCs, we performed an integrative analysis comparing active enhancer landscapes, transcriptional profiles, and functional genomics profiles of GSCs and non-neoplastic neural stem cells (NSCs). We identified sorting nexin 10 (SNX10), an endosomal protein sorting factor, as selectively expressed in GSCs compared with NSCs and essential for GSC survival. Targeting SNX10 impaired GSC viability and proliferation, induced apoptosis, and reduced self-renewal capacity. Mechanistically, GSCs utilized endosomal protein sorting to promote platelet-derived growth factor receptor β (PDGFRβ) proliferative and stem cell signaling pathways through posttranscriptional regulation of the PDGFR tyrosine kinase. Targeting SNX10 expression extended survival of orthotopic xenograft–bearing mice, and high SNX10 expression correlated with poor glioblastoma patient prognosis, suggesting its potential clinical importance. Thus, our study reveals an essential connection between endosomal protein sorting and oncogenic receptor tyrosine kinase signaling and suggests that targeting endosomal sorting may represent a promising therapeutic approach for glioblastoma treatment.

## Graphical abstract

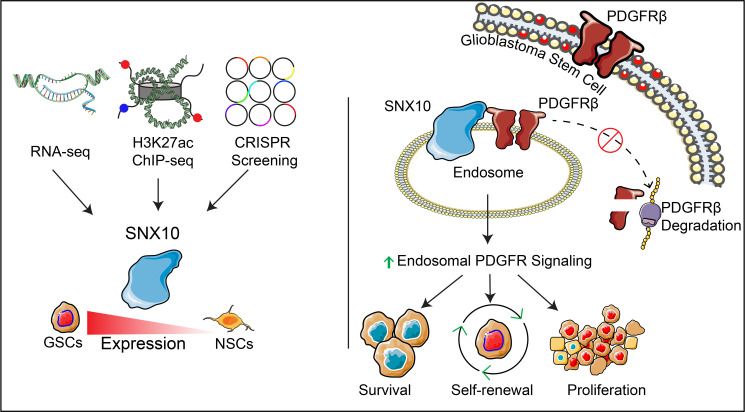

## Introduction

As the most prevalent primary intrinsic brain tumor, glioblastomas are highly lethal, with current standard-of-care therapies offering only palliation. Several factors contribute to poor prognosis, including intra- and intertumoral heterogeneity, invasion into normal brain, and universal recurrence following surgical resection (1, 2). The cancer stem cell hypothesis posits the presence of a stem-like tumor cell state to initiate tumors and recapitulate the cellular diversity of the tumor from which they were derived (3). Functionally defined glioblastoma stem cells (GSCs) (4) have been reliably identified in glioblastoma (5, 6) and contribute to chemoresistance (7), radioresistance (8), angiogenesis (9), invasion (10), self-renewal, and recurrence (11). Targeting both GSCs and differentiated tumor components will be essential for achieving more durable therapeutic responses in patients with glioblastoma (4, 12, 13). Although controversy regarding GSCs still exists, numerous lines of evidence point toward neural stem and progenitor cells as the cell of origin in glioblastoma, including single-cell sequencing (14), lineage tracing (15), and functional studies (16). A deeper understanding of the molecular alterations that distinguish neoplastic stem cells from normal stem cells (NSCs) will be essential to precisely identify and target cancer-specific vulnerabilities.

Using integrative epigenomic and transcriptomic analysis followed by unbiased functional dependency mapping, we identified endosomal protein sorting as an essential process in GSCs. Endocytosis is a multicomponent series of processes involving internalization of cell surface proteins, trafficking of these factors for recycling or degradation, and sorting to distinct cellular sites (17, 18). Endosomes control receptor tyrosine kinase (RTK) signaling strength, spatial and temporal restrictions, and internalization (19). RTK signaling is the most commonly altered pathway in glioblastomas, with genetic alterations occurring in 86% of tumors, including epidermal growth factor receptor (EGFR), erb-B2 receptor tyrosine kinase 2 (ERBB2), and platelet-derived growth factor receptor (PDGFR) (20). Oncogenic signal transduction is activated or enhanced not only through genetic lesions, but also through epigenetic dysregulation, posttranslational modifications, and genetic fusions. Recently, mislocalization of intracellular trafficking via endocytosis has emerged as an oncogenic mechanism. Constitutive activation of Ras-associated binding 35 (RAB35), a small GTPase endosomal trafficking factor, drives PDGFRα to endosomes to activate downstream phosphoinositide 3-kinase (PI3K)/AKT signaling (21). While endosomes had been previously considered simply as an intermediate step on the way toward degradation, endosomes can serve as a platform for RTK signaling, including EGFR (22), PDGFR (23), vascular endothelial growth factor receptor (VEGFR) (24), and insulin receptor (25), among others (26). Thus, identification of the mechanisms underlying endosomal growth factor receptor signaling may elucidate a broader spectrum of oncogenic signaling and enhance our capacity to target these nodes for therapeutic benefit.

To discover novel functional regulators in GSCs, we leveraged comparative transcriptional landscapes between GSCs and NSCs to inform CRISPR screening in GSCs, identifying sorting nexin 10 (SNX10) as essential for GSC growth through endosomal sorting of PDGFRβ.

## Results

### Identification of epigenetically and transcriptionally upregulated genes in GSCs.

**Figure 1:**
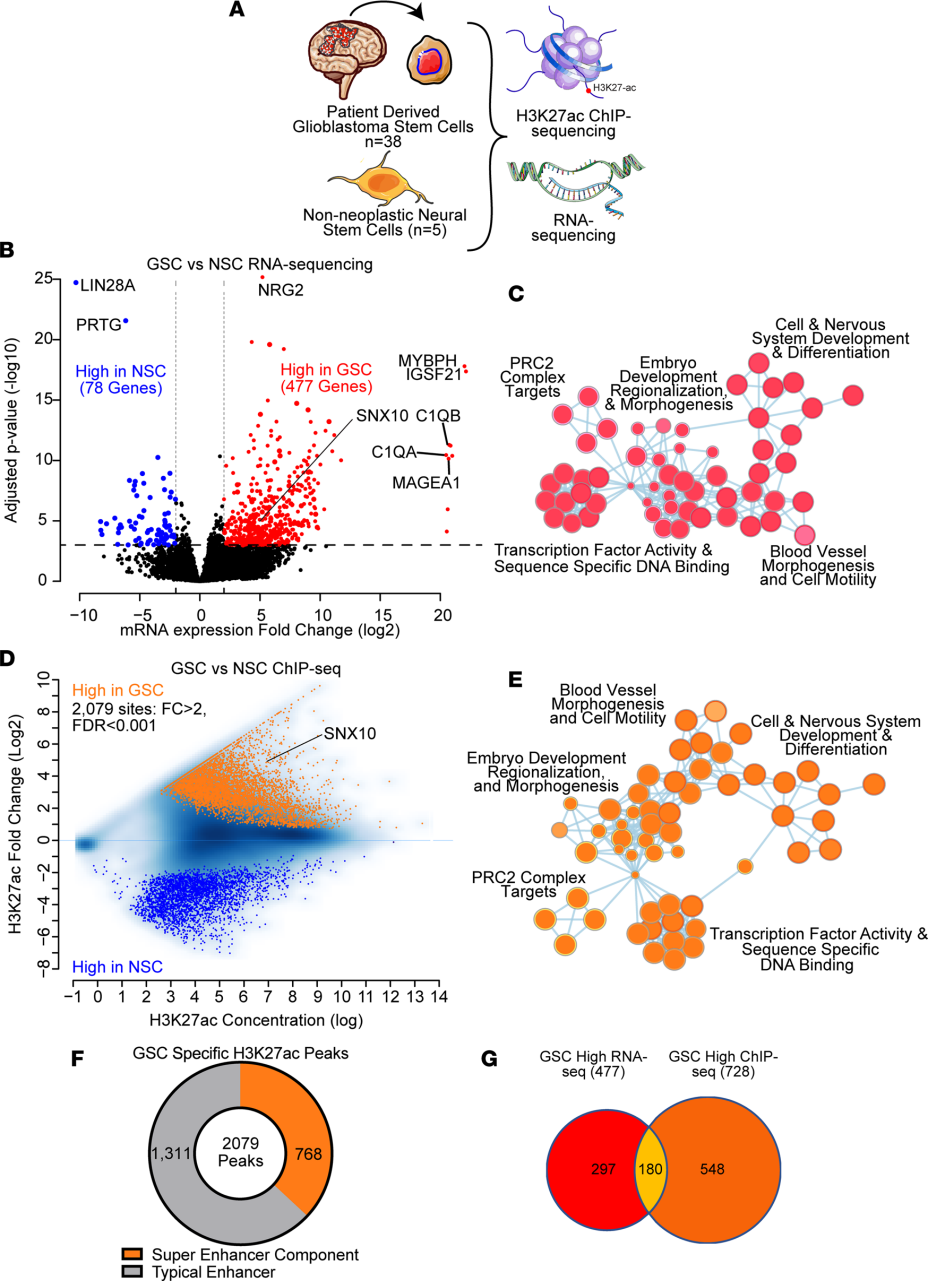
Integrated epigenetic and transcriptomic analysis of glioblastoma stem cells and non-neoplastic neural stem cells identified essential gene candidates. (**A**) Summary figure of RNA-seq and H3K27ac ChIP-seq performed in NSCs (*n* = 5) and patient-derived GSCs (*n* = 38). (**B**) Volcano plot showing differentially expressed genes by RNA-seq (27) between GSCs and NSCs. Differential expression cutoffs were (i) log_2_-transformed mRNA expression fold change greater than 2 and (ii) adjusted *P* value less than 1 × 10^–3^. (**C**) Gene set enrichment pathway connectivity diagram depicting gene sets enriched among genes upregulated in GSCs versus NSCs by RNA-seq. (**D**) MA plot showing differential H3K27ac ChIP-seq peaks (27) between GSCs (*n* = 38) and NSCs (*n* = 5). Differential peak cutoffs were (i) log_2_-transformed H3K27ac signal fold change greater than 2 and (ii) adjusted *P* value less than 1 × 10^–3^. (**E**) Gene set enrichment pathway connectivity diagram depicting gene sets enriched among genes upregulated in GSCs versus NSCs by H3K27ac ChIP-seq. (**F**) The fraction of GSC-specific H3K27ac peaks displayed in **D** that are components of super-enhancers versus typical enhancers. (**G**) Venn diagram overlap of genes upregulated in GSCs identified by both RNA-seq and ChIP-seq analysis in comparisons between GSCs and NSCs.

As GSCs and NSCs share numerous transcriptional programs, we hypothesized that genes differentially regulated at both enhancer and transcriptional activities between these 2 cell types may represent potential molecular targets with high therapeutic indices. To identify GSC-specific factors and processes, we performed integrative epigenetic and transcriptional analysis of 38 patient-derived GSCs and 5 NSCs (27) (Figure 1A). RNA-sequencing (RNA-seq) analysis using DESeq2 revealed 477 genes that were selectively upregulated in GSCs, while 78 were higher in NSCs (Figure 1B). Gene Ontology (GO) analysis indicated that genes upregulated in GSCs were enriched for processes that included transcription factor activity, polycomb repressive complex 2 (PRC2) targets, embryonic development, regionalization, and morphogenesis, and cell and nervous system development and differentiation (Figure 1C and Supplemental Figure 1A; supplemental material available online with this article; https://doi.org/10.1172/jci.insight.158077DS1). Profiling of the active enhancer and promoter landscapes of GSCs and NSCs through histone H3 lysine 27 acetyl chromatin immunoprecipitation followed by deep sequencing (H3K27ac ChIP-seq) uncovered 2,079 GSC-specific sites, of which 768 (37%) were a component of a super-enhancer in GSCs, and which mirrored gene ontology and developmental processes enriched through transcriptional analyses (Figure 1, D–F, and Supplemental Figure 1B). These findings suggest that GSCs may reactivate or repurpose developmental transcription factors. GSC-specific H3K27ac peaks contained motifs for a number of stem cell and developmental transcription factors, including grainyhead-like transcription factor 1 (GRHL1), double homeobox (DUX), SRY-box transcription factor 10 (SOX10), and neuronal differentiation 1 (NEUROD1) (Supplemental Figure 1C). Based on the hypothesis that genes dysregulated at both the epigenetic and transcriptional levels would be enriched for GSC-specific dependencies, overlap of genes upregulated at the transcriptional (*n* = 477) and epigenetic (*n* = 728, mapped from 2,079 H3K27ac ChIP-seq peaks) levels yielded 180 potential GSC-specific factors (Figure 1G), which were enriched in processes involved in epigenetic regulation, including PRC2 targets, and embryonic development including pattern specification and regionalization (Supplemental Figure 1, D and E). These 180 genes were prioritized for further analysis.

### Targeted CRISPR/Cas9 loss-of-function screen identified SNX10 as an essential target in GSCs.

**Figure 2:**
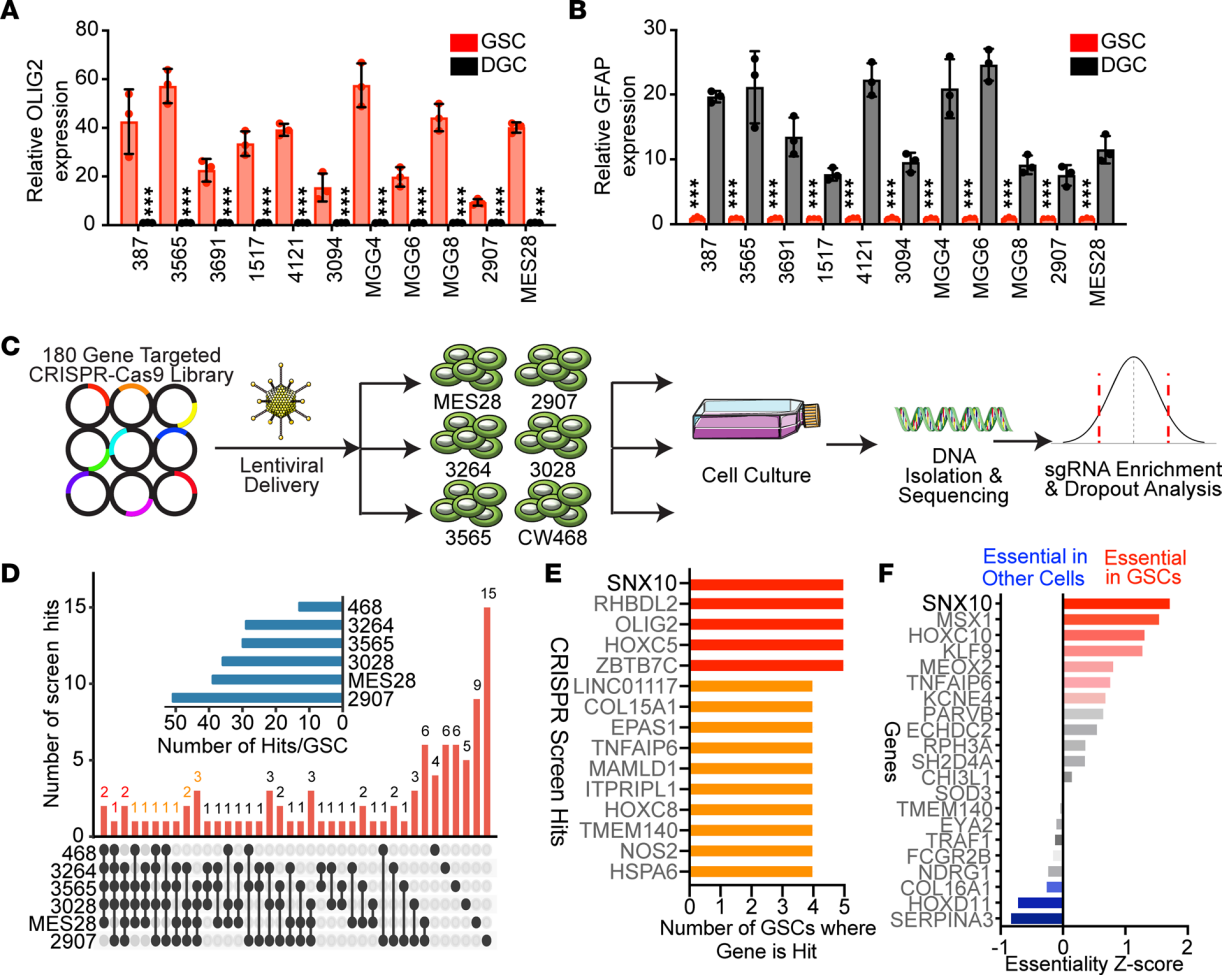
Targeted loss-of-function CRISPR dropout screen identified *SNX10* as a functional dependency in glioblastoma stem cells. (**A** and **B**) Relative mRNA expression of (**A**) *OLIG2* and (**B**) *GFAP* as assessed by qPCR in 11 matched GSCs relative to the paired differentiated glioma cell (DGC) sample. *n* = 3. Data are presented as mean ± SD. Significance was determined by 2-way ANOVA with Tukey’s multiple-comparison test. ***P < 0.001. (**C**) Schematic of loss-of-function CRISPR dropout screen of 180 genes in 6 patient-derived GSCs. (**D**) Overlap of essential genes in each GSC as defined by CRISPR screening. Six patient-derived GSCs were used in CRISPR screening experiments. Gene essentiality cutoffs were *z* score less than 0 and *P* value less than 0.05 for each GSC. (**E**) CRISPR screening hits ranked by the number of GSCs in which the gene was essential as defined in **D**. (**F**) Essentiality scores of CRISPR screening targets with patient prognostic significance in GSCs compared to other cancer cell lines in whole genome CRISPR screening data (34).

To assess the functional requirement for shared molecular targets identified above, we constructed a targeted CRISPR/Cas9 loss-of-function library against these 180 genes with 5 sgRNAs per gene and 100 nontargeting control sgRNAs, and transduced this library via lentivirus into 6 patient-derived GSCs representing 3 transcriptional subtypes (classical, proneural, and mesenchymal) (28). All GSCs used in this study were derived from human surgical resection specimens and have been previously functionally validated through their capacity to form tumors in serial transplantation assays and expressed high levels of the stem marker OLIG2 and low levels of the astrocyte differentiation marker GFAP compared with matched in vitro–differentiated glioma cells (DGCs) (Figure 2, A and B, Supplemental Figure 2A, and Tables 1 and 2). Following genomic editing and cell culture, DNA was isolated, and then sgRNA enrichment and dropout were assessed by next-generation sequencing (Figure 2C). Mapped reads for every sequenced sample were more than 300,000 (Supplemental Figure 2B), indicating that mapped reads for each sgRNA were more than 300. The distribution of counts indicated sufficient counts for sequencing (Supplemental Figure 2C). A Gini index of less than 0.1 (Supplemental Figure 2D) for each sample implied that our sgRNA read counts followed an even distribution and an index of 3 signified proper quality control during the screening process (29). Unique gene hits were identified in each GSC individually and overlap analyses were performed to identify common hits across multiple GSCs (Figure 2D). Top sgRNAs depleted from cells mapped to genes previously implicated in glioblastoma biology, including *OLIG2*, *NOS2*, *EPAS1* (HIF2A), and *AGAP-AS1* (30–33), validating our screening technique and approach (Supplemental Figure 2E). Five genes were essential in 5 out of 6 GSCs tested, including *ZBTB7C*, *RHBDL2*, *OLIG2*, *HOXC5*, and *SNX10* (Figure 2, D and E). We confirmed the functional importance of several of the screening hits using individually cloned sgRNAs against *OLIG2*, *NOS2*, *AKR1B10*, and *HOXA7* in GSCs and observed reduction in cell growth following targeting of these genes compared with a nontargeting sgRNA control (Supplemental Figure 3, A and B). Of the 5 common hits, *SNX10* was most highly correlated with poor glioblastoma patient prognosis in clinical data sets when overexpressed and demonstrated GSC-specific essentiality when we interrogated a previously published in silico data set (34) (Figure 2F). Collectively, SNX10 represents a potentially selective dependency in GSCs.

### GSCs preferentially express SNX10.

**Figure 3:**
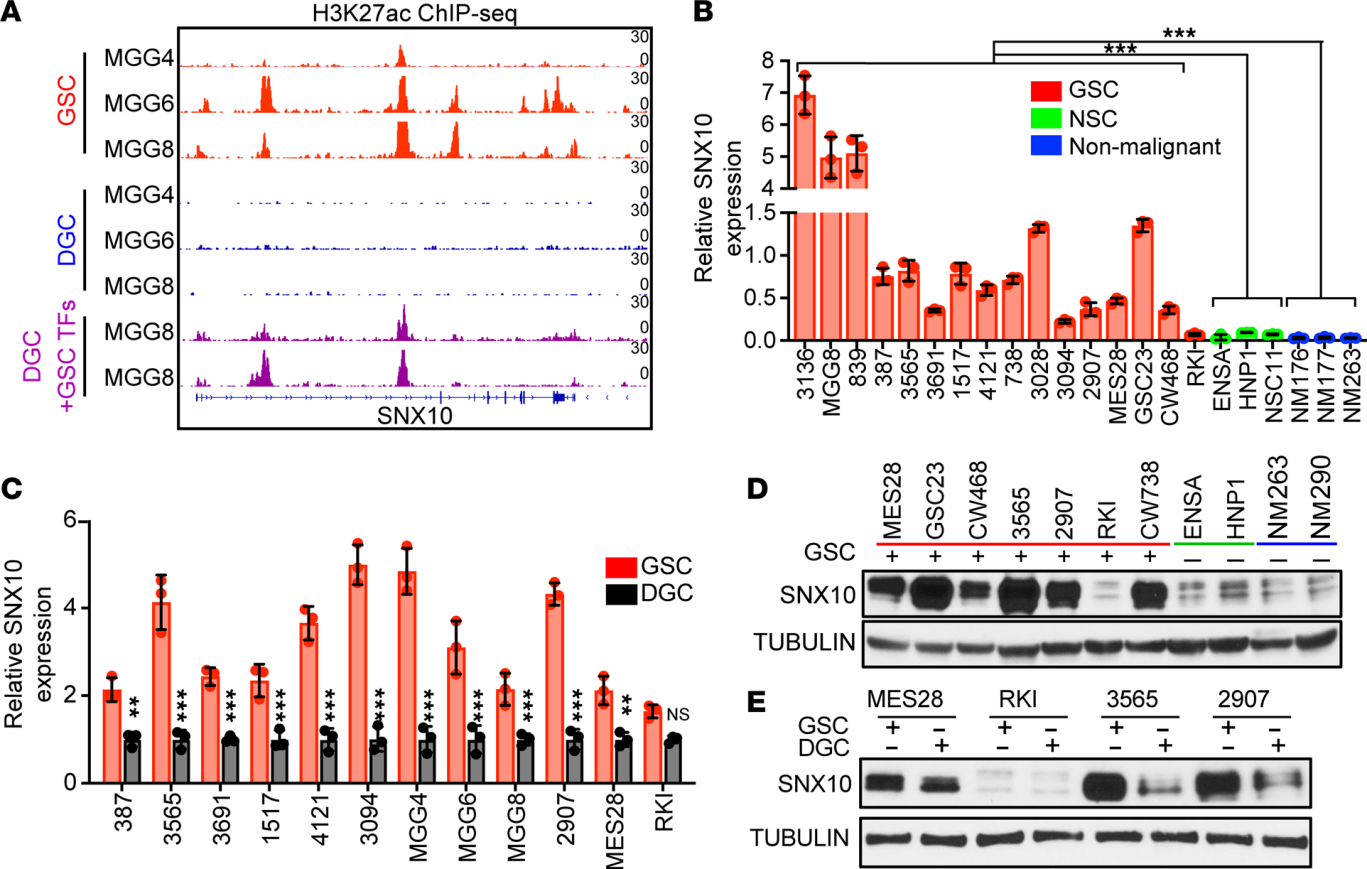
SNX10 is preferentially expressed in glioblastoma stem cells compared with non-neoplastic neural stem cells, nonmalignant neural cultures, and differentiated glioblastoma cells. (**A**) H3K27ac ChIP-seq signal across the *SNX10* locus in 3 matched GSC (red), differentiated glioma cell (DGC) samples (blue), and 2 replicates of DGCs reprogrammed with GSC-specific transcription factors (TFs, purple) (35). (**B**) qPCR analysis of *SNX10* mRNA expression in different GSCs, NSCs, and nonmalignant (NM) brain cultures. *n* = 3. (**C**) qPCR analysis of *SNX10* mRNA expression in GSCs and matched DGCs. *n* = 3. (**D**) Western blot of SNX10 protein levels in different GSCs (marked in red), NSCs (marked in green), and nonmalignant (marked in blue) brain culture. (**E**) Western blot to examine SNX10 protein levels in different GSCs and matched DGCs. In **D** and **E**, tubulin was used as loading control. Samples were run in a single gel with loading controls shown in the supplemental material. Data are presented as mean ± SD. *P* values were calculated by 2-way ANOVA with Tukey’s multiple-comparison test. **P < 0.01; ***P < 0.001. NS, not significant.

*SNX10* was identified as a top candidate gene required for GSC proliferation in the loss-of-function CRISPR/Cas9 screen. To better understand the regulation of SNX10 expression in GSCs, we examined the *SNX10* promoter and enhancer landscapes in GSCs and NSCs, revealing that the *SNX10* locus demonstrated enrichment for H3K27ac signals in GSCs compared with NSCs (Supplemental Figure 3C). *SNX10* displayed high promoter and enhancer H3K27ac signals in GSCs compared with matched serum-differentiated DGCs and the H3K27ac signal was partially rescued through forced expression of GSC reprogramming factors in differentiated cells (Figure 3A) (35). *SNX10* mRNA expression was upregulated across a panel of patient-derived GSCs from various transcriptional subtypes compared with NSCs by RNA-seq, which we validated by qPCR in GSCs, NSCs, and nonmalignant brain cell cultures derived from epilepsy surgical specimens (Figure 3B). In a large panel of matched GSC-DGC pairs, *SNX10* expression was consistently elevated at the mRNA level measured by qPCR (Figure 3C). Concordantly, SNX10 protein levels were elevated in GSCs compared with both NSCs or nonmalignant cultures (Figure 3D), as well as in GSCs compared with DGCs (Figure 3E). Taken together, these results show that GSCs preferentially express SNX10 compared with nonneoplastic neural cells or differentiated tumor cells.

### SNX10 is essential for GSC proliferation and survival.

**Figure 4:**
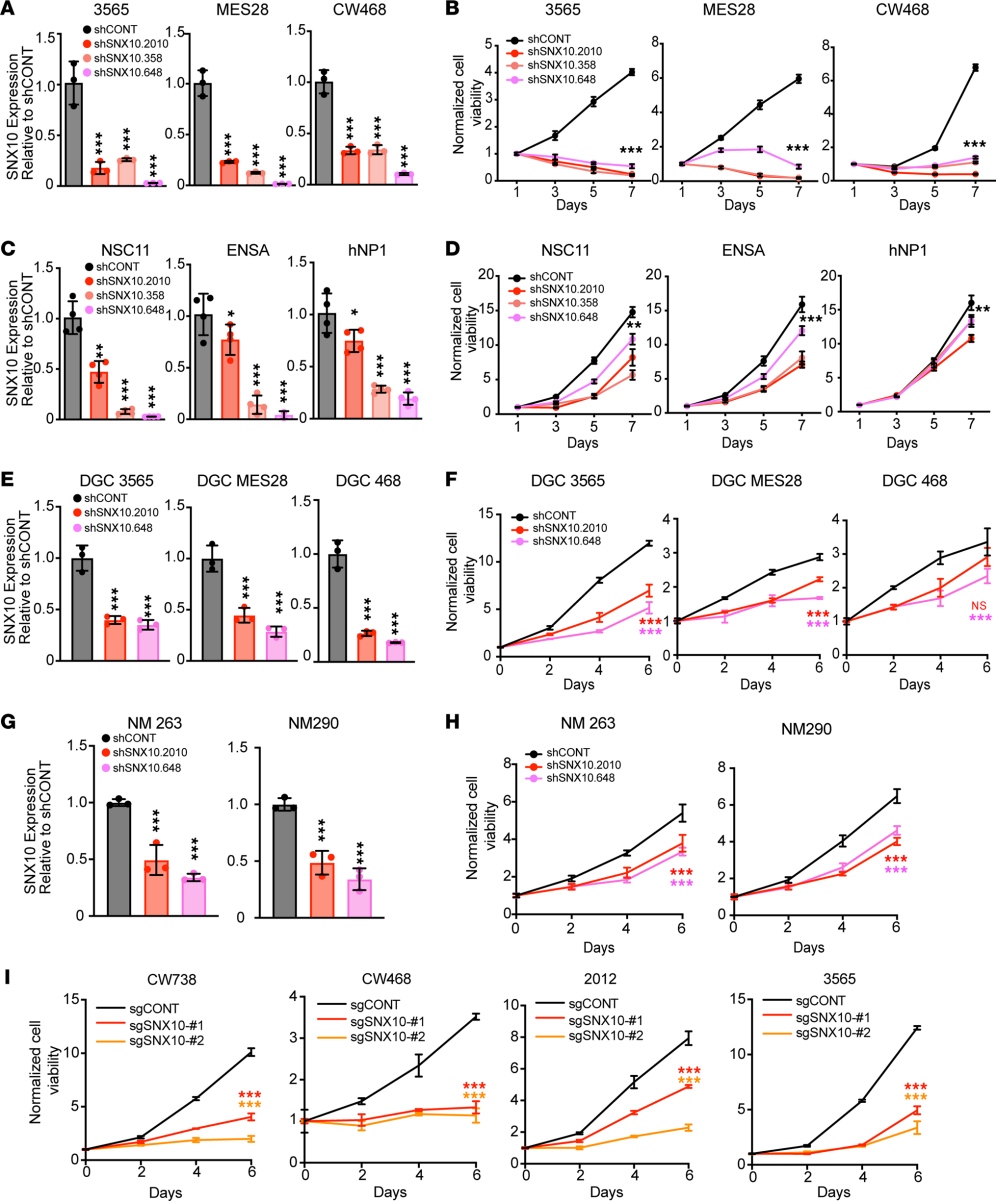
SNX10 preferentially affects glioblastoma stem cell proliferation and survival. (**A**) *SNX10* mRNA expression by qPCR in 3 patient-derived GSCs following transduction with 3 independent nonoverlapping shRNAs targeting SNX10 or a nontargeting control shRNA (shCONT). *n* = 3. *P* values were calculated by 1-way ANOVA with Tukey’s multiple-comparison test. ***P < 0.001. (**B**) Normalized cell viability of 3 patient-derived GSCs following transduction with 3 shRNAs targeting SNX10 compared to shCONT over a 7-day time course. Repeated measures 2-way ANOVA with Dunnett’s multiple test correction was used for statistical analysis. ***P < 0.001 for all shSNX10 versus shCONT comparisons. (**C**) *SNX10* mRNA expression by qPCR in 3 NSCs following transduction with 3 independent nonoverlapping shRNAs targeting SNX10 or shCONT. *n* = 4. *P* values were calculated by 1-way ANOVA with Tukey’s multiple-comparison test. *P < 0.05; **P < 0.01; ***P < 0.001. (**D**) Normalized cell viability of 3 NSCs following transduction with 3 shRNAs targeting SNX10 compared to shCONT over a 7-day time course. Repeated measures 2-way ANOVA with Dunnett’s multiple test correction was used for statistical analysis. **P < 0.01; ***P < 0.001 for shSNX10 versus shCONT comparisons. (**E**) Relative *SNX10* mRNA expression in 3 DGCs following transduction of 2 shRNAs targeting SNX10 compared to shCONT. *n* = 3. Significance was determined by 1-way ANOVA with Tukey’s multiple-comparison test. (**F**) Normalized cell viability of 3 DGCs following transduction with 2 shRNAs targeting SNX10 compared to shCONT over a 6-day time course. *n* = 3. Significance was determined by 2-way ANOVA with Tukey’s multiple-comparison test. ***P < 0.001. (**G**) Relative *SNX10* mRNA expression in nonmalignant brain culture 263 (NM263) or NM290 following transduction of 2 shRNAs targeting SNX10 compared to shCONT. *n* = 3. Significance was determined by 1-way ANOVA with Tukey’s multiple-comparison test. ***P < 0.001. (**H**) Normalized cell viability of NM263 or NM290 following transduction with 2 shRNAs targeting SNX10 compared to shCONT over a 6-day time course. *n* = 3. Significance was determined by 2-way ANOVA with Tukey’s multiple-comparison test. ***P < 0.001 for all shSNX10 versus shCONT comparisons. (**I**) Normalized cell viability of GSC CW738, GSC CW468, GSC 2012, and GSC 3565 following transduction with 1 of 2 sgRNAs targeting SNX10 compared to a nontargeting sgRNA (sgCONT) over a 6-day time course. *n* = 3. Significance was determined by 2-way ANOVA with Tukey’s multiple-comparison test. Data are presented as mean ± SD. NS, not significant.

**Figure 5:**
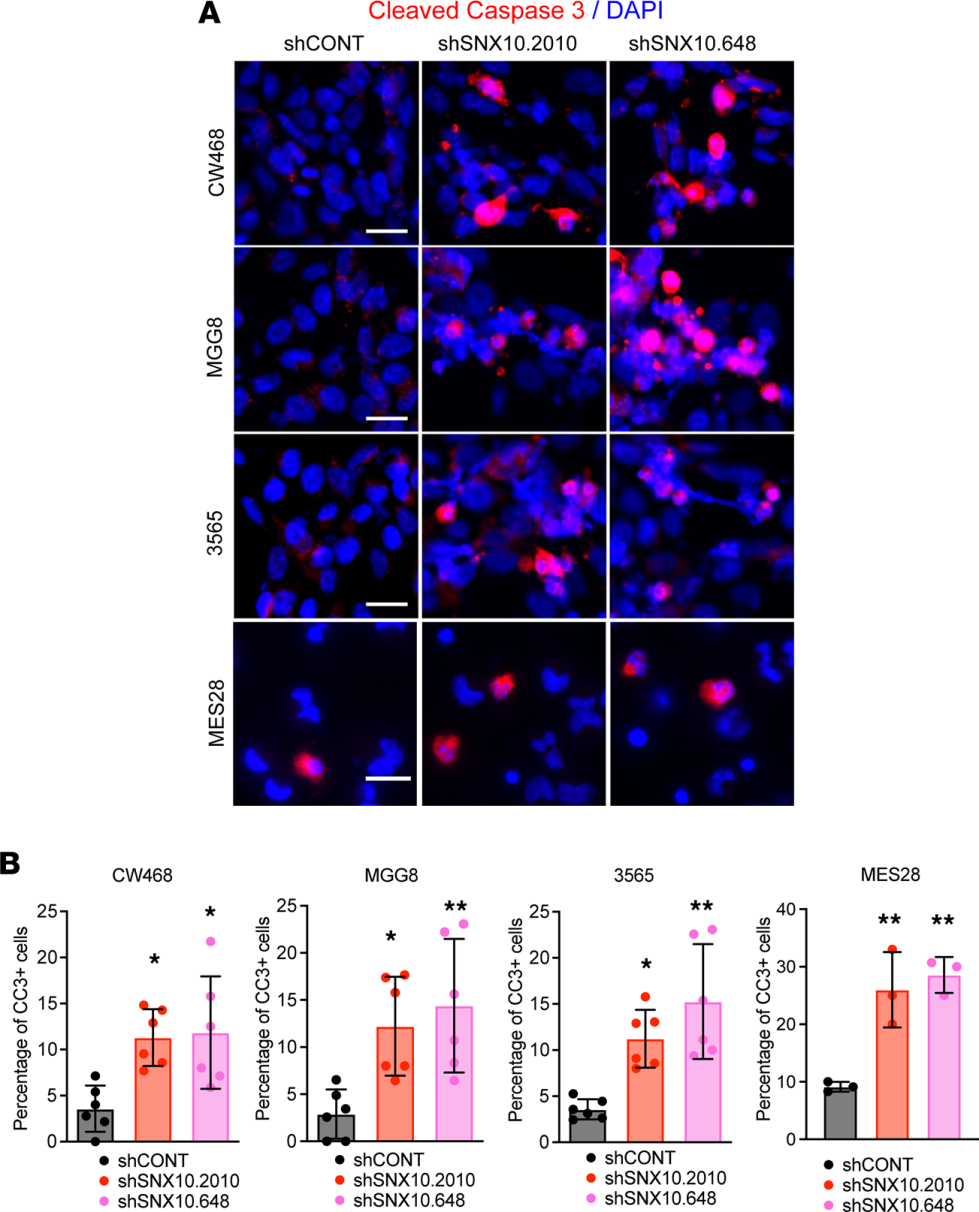
SNX10 is essential for glioblastoma stem cell survival. (**A**) Immunofluorescent staining of cleaved caspase 3 (CC3) in GSCs transduced with shCONT or shSNX10. CC3 is shown in red; DAPI in blue. Scale bars: 20 μm. (**B**) Quantification of CC3-positive cells in GSCs transduced with shCONT or shSNX10. *n* = 3. Data are presented as mean ± SD. Significance was determined by 1-way ANOVA with Tukey’s multiple-comparison test. *P < 0.05; **P < 0.01.

To interrogate the functional importance of SNX10 in GSCs, we performed knockdown experiments using 3 independent nonoverlapping shRNAs compared to a nontargeting control shRNA encoding a sequence not expressed in the mammalian genome (shCONT). SNX10 knockdown impaired GSC growth across a panel of different patient-derived GSCs (Figure 4, A and B, and Supplemental Figure 4, A and B), while the effects generated by SNX10 knockdown in NSCs were less significant than that in GSCs (Figure 4, C and D, and Supplemental Figure 4C). SNX10 knockdown only mildly reduced cell growth in DGCs (Figure 4, E and F, and Supplemental Figure 4D) and in nonmalignant brain cell cultures (Figure 4, G and H). Using an orthogonal approach, CRISPR/Cas9 deletion of *SNX10* similarly impaired cell proliferation in 4 patient-derived GSCs (Figure 4I). Consistently, SNX10 knockdown with shRNA induced apoptosis measured by cleaved caspase 3 immunofluorescent staining in GSCs (Figure 5, A and B), which was confirmed by Annexin V/propidium iodide staining via flow cytometry (Supplemental Figure 4, E and F). Thus, SNX10 is a GSC-specific vulnerability for cell proliferation and survival relative to nonneoplastic NSCs, differentiated tumor cells, and nonmalignant neural cultures.

### SNX10 maintains GSC cell cycle, stem cell programs, and self-renewal.

**Figure 6:**
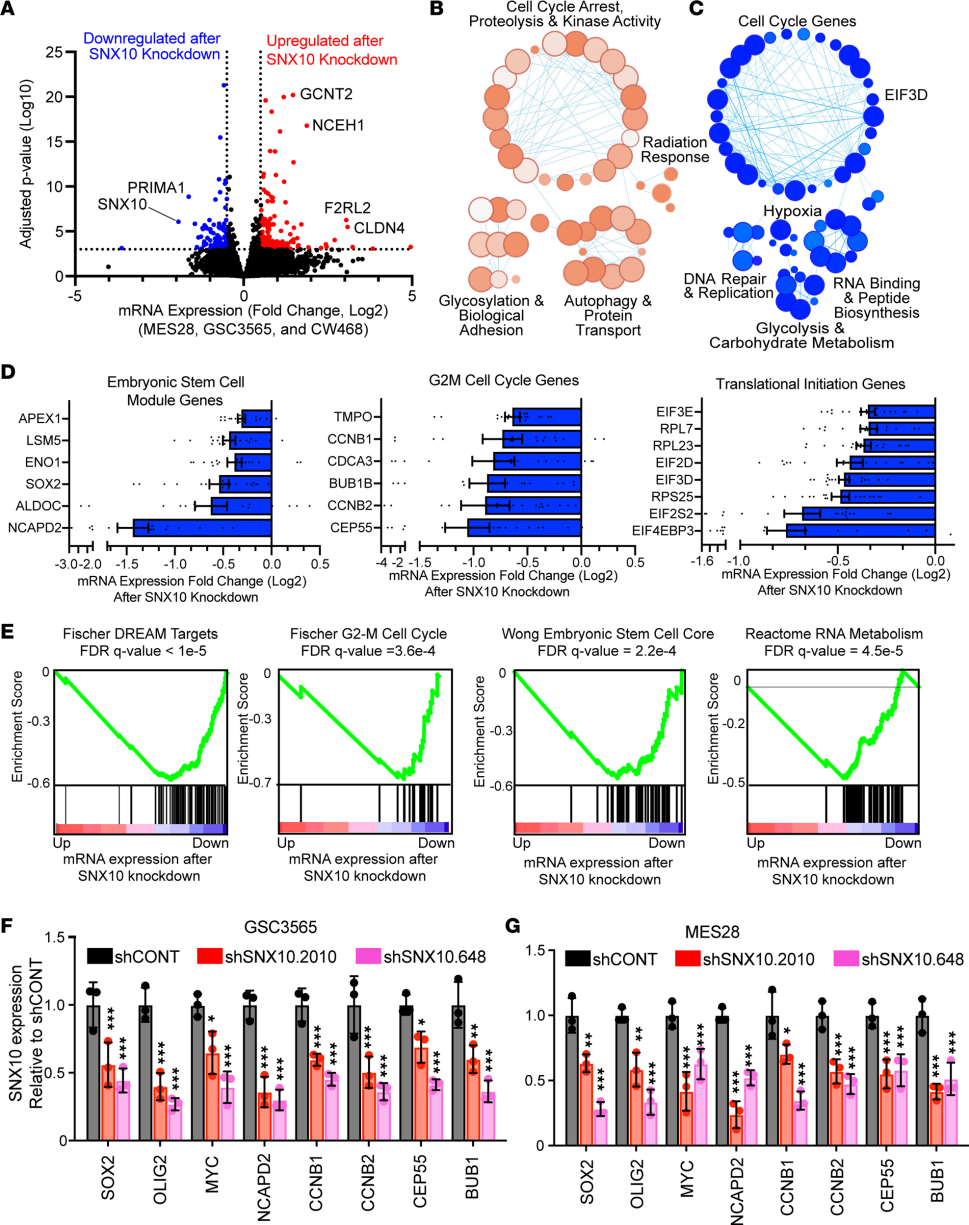
SNX10 is required for GSC maintenance by regulating stem cell programs and cell cycle progression. (**A**) Volcano plot of differentially expressed genes between cells transduced with 3 independent nonoverlapping shRNAs targeting SNX10 versus cells transduced with a nontargeting control shRNA (shCONT) in 3 patient-derived GSCs. Red indicates genes upregulated following SNX10 knockdown and blue indicates genes downregulated following SNX10 knockdown. (**B** and **C**) Gene set enrichment pathway connectivity diagram depicting gene sets enriched among genes (**B**) upregulated and (**C**) downregulated following SNX10 knockdown as indicated in **A**. (**D**) mRNA expression fold change of selected genes following SNX10 knockdown in 3 patient-derived GSCs relative to shCONT in selected gene sets as measured by RNA-seq. Error bars indicate SEM. (**E**) GSEA of gene sets downregulated following SNX10 knockdown by RNA-seq. FDR *q* value was calculated for statistical analysis. (**F** and **G**) mRNA expression of stemness genes and cell cycle genes following SNX10 knockdown in (**F**) GSC 3565 and (**G**) GSC MES28. *n* = 3. Data are presented as mean ± SD. Significance was determined by 2-way ANOVA with Tukey’s multiple-comparison test. *P < 0.05; **P < 0.01; ***P < 0.001.

**Figure 7:**
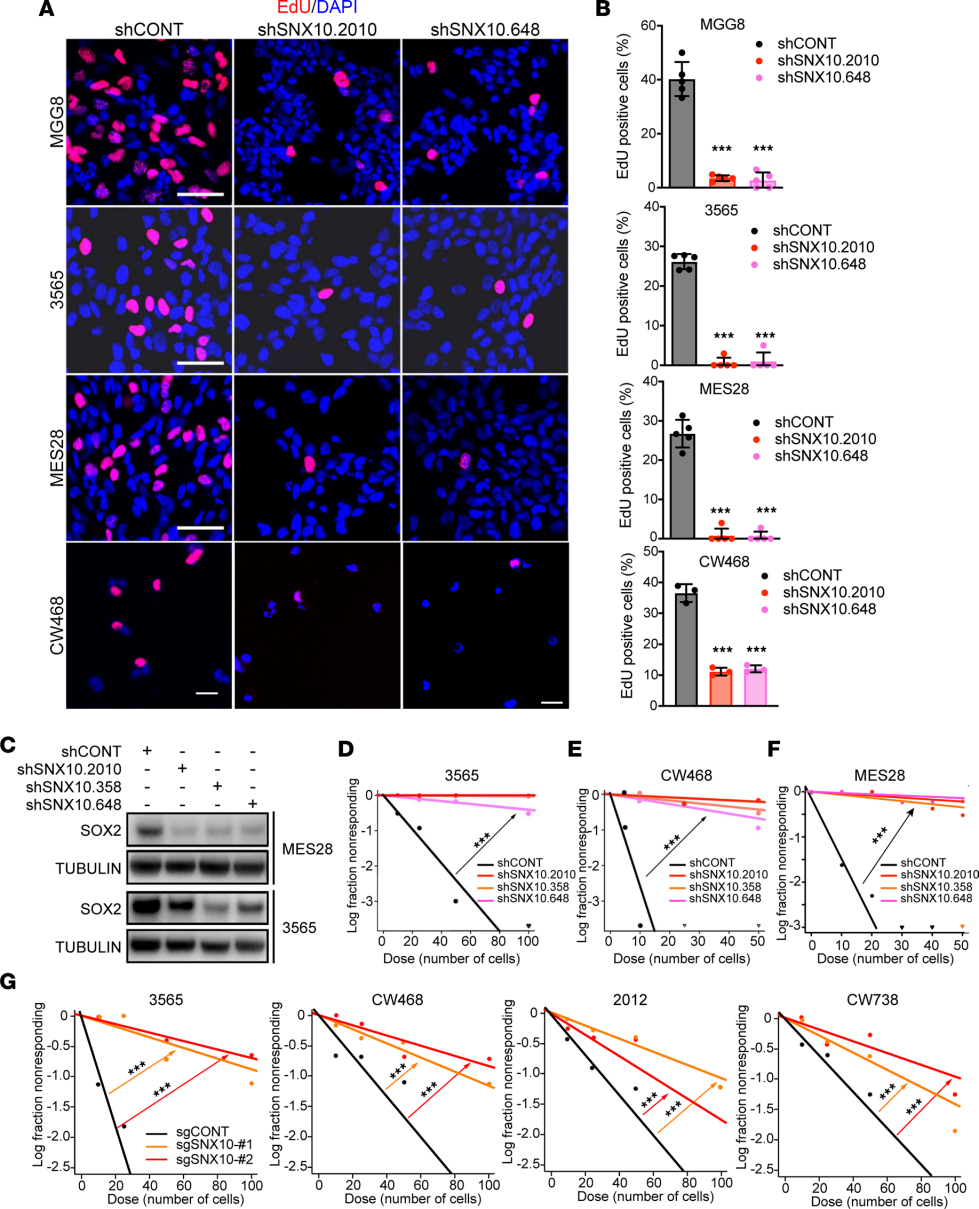
SNX10 is essential for maintenance of stemness and self-renewal capacity in glioblastoma stem cells. (**A**) EdU staining in different GSCs transduced with shRNAs targeting SNX10 expression or a nontargeting shRNA (shCONT). EdU is in red, DAPI in blue. Scale bars: 20 μm. (**B**) Quantification of EdU-positive cells in GSCs transduced with shCONT or shSNX10. *n* = 3. (**C**) Protein levels of SOX2 in 2 patient-derived GSCs following transduction with 3 independent nonoverlapping shRNAs targeting SNX10 or shCONT. Tubulin was used as a loading control. Samples were run in a single gel with loading controls shown in supplemental material. (**D–F**) Limiting dilution assay (LDA) in (**D**) GSC 3565, (**E**) GSC 468, and (**F**) GSC MES28 following transduction with 1 of 3 shRNAs targeting SNX10 compared to shCONT. (**G**) LDA in GSC 3565, GSC CW468, GSC 2012, or GSC CW738, following transduction with 1 of 2 sgRNAs targeting SNX10 compared to a nontargeting sgRNA (sgCONT). Data in panel **B** are presented as mean ± SD. Significance was determined by 1-way ANOVA with Tukey’s multiple-comparison test. For panels **D**–**G**, was determined using extreme limiting dilution assays as described in Hu and Smyth (79). ***P < 0.001.

To define the mechanism of action of SNX10 in GSCs, we performed transcriptional profiling through RNA-seq following knockdown of SNX10 with 3 independent nonoverlapping shRNAs in 3 patient-derived GSCs (MES28, GSC 3565, and CW468) (Figure 6A). Pathways involved in cell cycle arrest, radiation response, protein transport, and autophagy were upregulated after SNX10 knockdown (Figure 6B). Specifically, gene sets involved in regulation of chaperone-mediated autophagy, including the individual genes *LAMP2* and *CTSA*, were upregulated following SNX10 knockdown, consistent with prior reports (36, 37). Conversely, pathways involved in cell cycle, DNA repair and replication, glycolytic metabolism, and RNA binding and peptide biosynthesis were downregulated (Figure 6C). Specifically, DREAM complex factors that regulate cell cycle–dependent gene expression and balance cellular quiescence and proliferation (38), signatures of the G_2_/M cell cycle checkpoint, core stem cell genes, and RNA metabolism genes were downregulated after SNX10 knockdown (Figure 6, D and E). These findings were validated by qPCR, with consistent reductions in selected stem cell and cell cycle genes (Figure 6, F and G). SNX10 knockdown in GSCs impaired cellular proliferation and altered the cell cycle profile in 5-ethynyl-2′-deoxyuridine (EdU) incorporation assays (Figure 7, A and B), supporting the changes in cell cycle transcriptional programs in GSCs upon SNX10 knockdown.

Previous reports have found that endosome/lysosome homeostasis is essential for the survival and stemness of GSCs by regulating the mTOR pathway (39). SNX10 plays a crucial role in endosome/lysosome homeostasis by sorting cargo as a member of the sorting nexin family of proteins (40). Therefore, we next investigated the role of SNX10 in the maintenance of stemness. Knockdown of SNX10 reduced mRNA expression levels of critical GSC stemness factors, SOX2 and OLIG2, and reduced SOX2 protein expression in GSCs (Figure 7C and Supplemental Figure 5, A and B). SNX10 depletion reduced sphere formation capacity of GSCs measured by in vitro limiting dilution assay (LDA), demonstrating that SNX10 knockdown functionally impairs stemness of GSCs (Figure 7, D–F). Confirming the functional importance of SNX10 in maintenance of GSC stemness, SNX10 knockout with CRISPR/Cas9 phenocopied knockdown studies with impaired self-renewal capacity, indicating a loss of stemness in GSCs (Figure 7G) and reduced SOX2 protein levels (Supplemental Figure 5C). Altogether, these results indicate that SNX10 is essential for GSC proliferation, cell cycle progression, and stemness.

### SNX10 promotes endosomal growth factor receptor signaling in GSCs.

**Figure 8:**
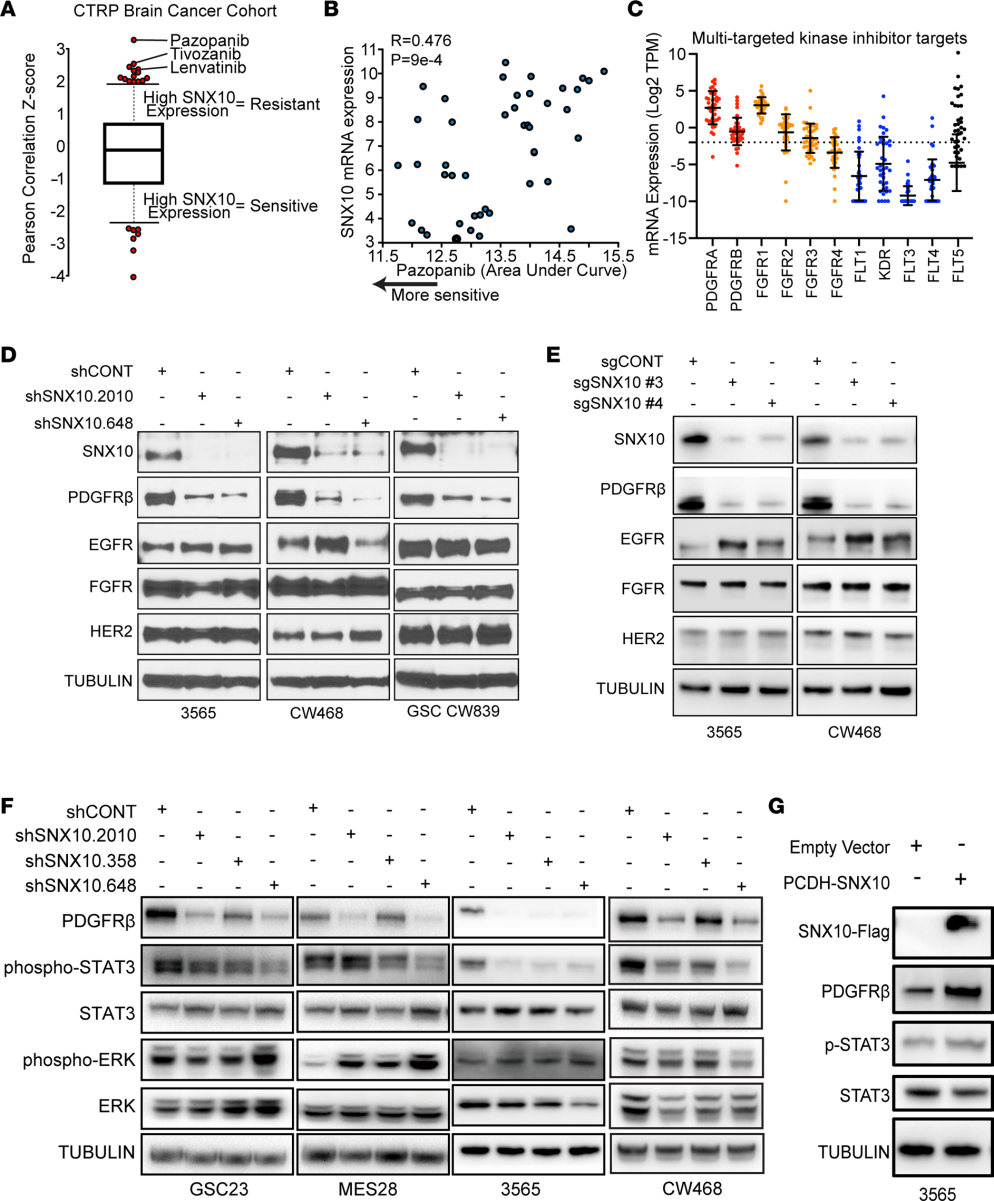
SNX10 supports endosomal PDGFR signaling in GSCs. (**A**) Therapeutic efficacy prediction of drugs in all brain cancer cells in the Cancer Response Therapeutics Portal (CTRP) v2 data set based on *SNX10* mRNA expression. Positive correlation *z* score indicates that high *SNX10* mRNA expression is associated with resistance to the listed drug. (**B**) Correlation between pazopanib area under the curve (AUC) and *SNX10* mRNA expression in all brain cancer cells in the CTRP v2 data set. Each dot indicates an individual brain cancer cell line. Pearson’s correlation coefficient (*r*) is 0.476. Pearson’s correlation for the *z* score is 3.31. The *P* value is 9 × 10^–4^. (**C**) mRNA expression by RNA-seq of multitargeted kinase inhibitors indicated in **B** in a panel of 38 GSCs. Each dot indicates an individual patient-derived GSC. (**D**) Western blot showing protein levels of selected receptor tyrosine kinases in GSC 3565, GSC CW468, and GSC CW839 following transduction with 1 of 2 shRNAs targeting SNX10 or a nontargeting shRNA (shCONT). Tubulin was used as a loading control. Samples were run contemporaneously in separate gels, with individual loading controls shown in the supplemental material. (**E**) Western blot showing protein levels of selected receptor tyrosine kinases in GSC 3565 and GSC CW468 following transduction with 1 of 2 CRISPR/Cas9-mediated sgRNAs targeting SNX10 compared to a nontargeting sgRNA (sgCONT). Tubulin was used as a loading control. Samples were run in a single gel with entire gels shown in the supplemental material. (**F**) Western blot showing protein levels of PDGFRβ, phospho-STAT3, phospho-ERK, total STAT3, and total ERK in GSC 23, GSC MES28, GSC 3565, and GSC CW468 following transduction with 1 of 2 shRNAs targeting SNX10 or shCONT. Tubulin was used as a loading control. Samples were run contemporaneously in separate gels, with individual loading controls shown in the supplemental material. (**G**) Western blot showing protein levels of FLAG-tagged SNX10, PDGFRβ, phospho-STAT3, and STAT3 in GSC 3565 following transduction with an SNX10 overexpression construct. Tubulin was used as a loading control. Samples were run in a single gel, with entire gels shown in the supplemental material.

Endosomal protein sorting serves essential roles in cancer biology through proper localization of proteins, autophagy, and lysosomal degradation. To understand the role of SNX10 in GSC biology, we leveraged the Cancer Therapeutics Response Portal (CTRP) (41, 42), in which cellular proliferation responses to 481 compounds across 860 cancer cell lines were tested with gene expression data. Within brain cancer cell lines in this cohort, high *SNX10* expression was associated with resistance to a number of multitargeted RTK inhibitors, including pazopanib, tivozanib, and lenvatinib, which each target VEGFRs, PDGFRs, FGFRs, and KIT with varying selectivity (Figure 8, A and B). Of the molecular targets of these agents, PDGFRα, PDGFRβ, and FGFR1 had the highest expression in GSCs, supporting potential roles as selective drug targets, while expression of VEGFRs (encoded by *FLT1*, *KDR*, *FLT3*, and *FLT4*) had low-to-undetectable levels in GSCs (Figure 8C). We hypothesized that SNX10 may have a role in regulation of these RTKs. Knockdown of SNX10 with shRNAs selectively reduced PDGFRβ protein levels, while the levels of related RTKs, EGFR, HER2, and FGFR, were relatively unchanged (Figure 8D). The loss of PDGFRβ following SNX10 depletion was consistent across multiple GSCs measured in orthogonal approaches by both shRNA-mediated knockdown and CRISPR/Cas9-mediated knockout (Figure 8, D and E), suggesting that SNX10 is involved in the regulation of PDGFRβ expression.

**Figure 9:**
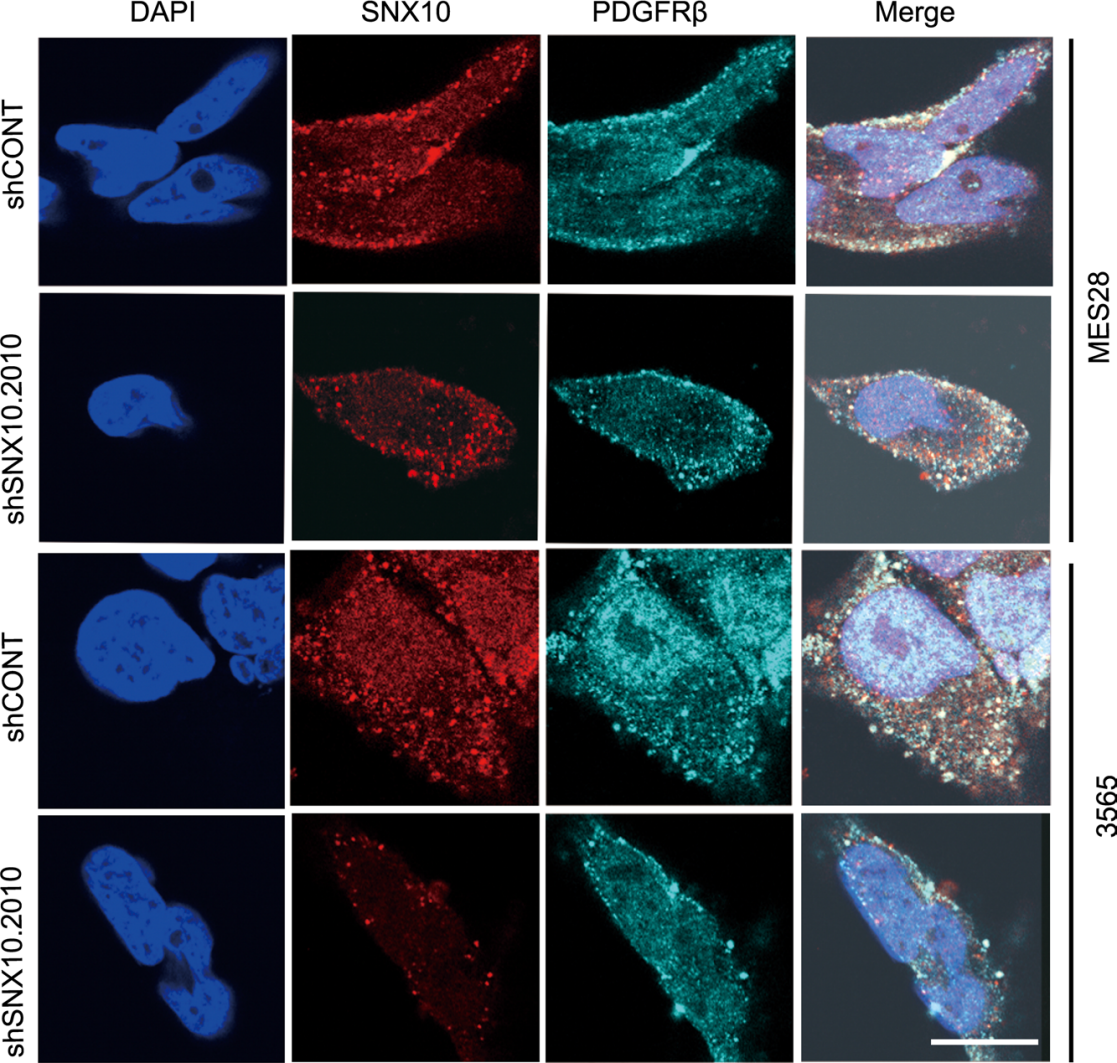
SNX10 colocalizes with PDGFRβ in glioblastoma stem cells. (**A**) Immunofluorescent staining of SNX10 and PDGFRβ protein levels and localization after SNX10 knockdown in GSC 3565 and MES28. Scale bar: 10 μm.

We next investigated the effects of targeting SNX10 on downstream PDGFRβ signaling, which occurs through several downstream elements. STAT3 phosphorylation, a readout for endosomal PDGFR signaling (23, 43, 44), was downregulated upon SNX10 knockdown with shRNAs or upon knockout with CRISPR/Cas9 in GSCs, while other downstream effectors, ERK and AKT, were unaffected (Figure 8F and Supplemental Figure 5, D and E). Reciprocally, SNX10 overexpression enhanced STAT3 phosphorylation and PDGFRβ protein levels (Figure 8G). Moreover, coimmunostaining of SNX10 and PDGFRβ in MES28 and GSC 3565 cells showed that SNX10 colocalized with PDGFRβ and knockdown of SNX10 decreased the protein level of PDGFRβ (Figure 9A).

**Figure 10:**
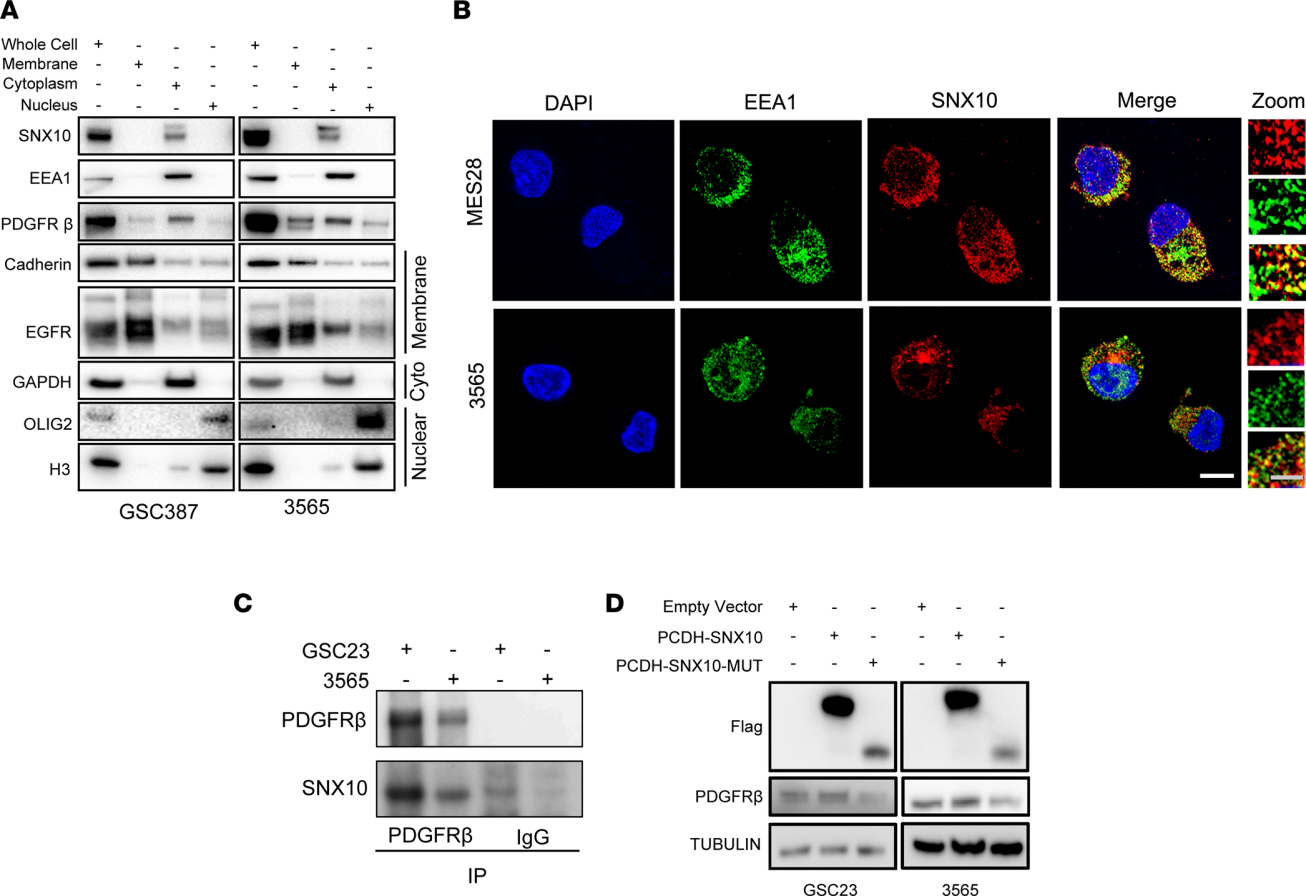
SNX10 interacts with PDGFRβ in the endosomal compartment via its phox homology domain. (**A**) Western blot of SNX10 and PDGFRβ in GSC 387 and GSC 3565 following cell fractionation. Pan-cadherin and EGFR were used as membrane markers, GAPDH was used as a cytoplasmic marker, and OLIG2 and histone H3 were used as nuclear markers. EEA1 was used as an early endosome marker. Samples were run contemporaneously in separate gels with whole gels shown in the supplemental material. (**B**) Immunofluorescent staining of EEA1 and SNX10 in MES28 and 3565. Scale bars: 10 μm and 5 μm (zoomed-in images). (**C**) Western blot showing immunoprecipitation with pulldown of PDGFRβ in GSC 3565 and GSC 23 compared to a nonspecific IgG control. Immunoblotting was performed with anti-PDGFRβ and -SNX10 antibodies. Samples were run in a single gel with entire gels shown in the supplemental material. (**D**) Western blot showing protein levels of FLAG, PDGFRβ, and tubulin after transduction with PCDH-SNX10 or PCDH-SNX10MUT (PX-domain deficient). Samples were run in a single gel with entire gels shown in the supplemental material.

To explore the role of SNX10 in regulating PDGFRβ expression, we performed cell fractionation experiments in different GSCs and found that SNX10 colocalized with PDGFRβ in the cytoplasmic fraction, marked by the early endosome marker EEA1, suggesting that SNX10 may regulate PDGFRβ expression via endosome sorting (Figure 10A). Immunofluorescent staining showed that SNX10 colocalized with endosome marker EEA1 (Figure 10B). Additionally, immunoprecipitation of PDGFRβ enriched for SNX10, indicating a physical interaction between these factors, either directly or as part of a complex (Figure 10C). The phox homology (PX) domain has been reported to be essential for the vacuolation activity of SNX10 (45), and we hypothesized that this domain would be important for mediating the interaction between SNX10 and PDGFRβ. Thus, we overexpressed full-length and PX-domain-deleted SNX10 in GSCs and assessed PDGFRβ protein levels. Overexpression of the PX-domain-deleted SNX10 construct did not increase protein levels of PDGFRβ, suggesting an important role for this domain in the control of PDGFRβ expression (Figure 10D).

**Figure 11:**
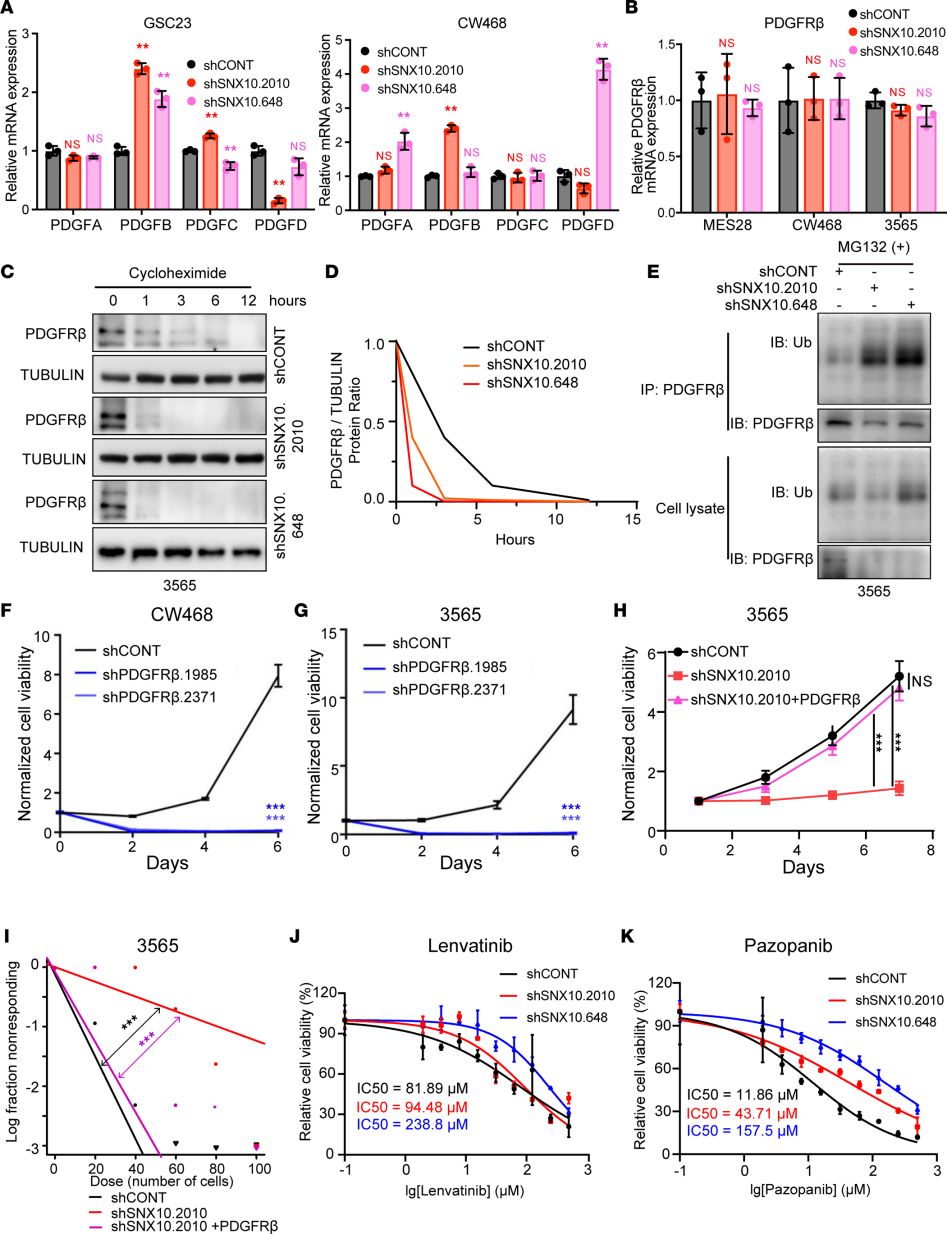
SNX10 maintains PDGFRβ protein stability via a posttranscriptional mechanism and alters sensitivity to multitargeted kinase inhibitors. (**A**) qPCR analysis of PDGFA–D mRNA expression in 2 GSCs after SNX10 knockdown. mRNA expression was normalized to actin. (**B**) qPCR analysis of PDGFRβ mRNA expression in 3 GSCs after SNX10 knockdown. mRNA expression was normalized to actin. (**C**) Western blot showing protein levels of PDGFRβ in GSC 3565 following transduction with 1 of 2 shRNAs targeting SNX10 or a nontargeting shRNA (shCONT) over a 12-hour time course following treatment with cycloheximide (10 μg/mL). Samples were run in a single gel, with entire gels shown in the supplemental material. (**D**) Quantification of PDGFRβ density (relative to tubulin) showing protein degradation rate of PDGFRβ in GSC 3565 following transduction with 1 of 2 shRNAs targeting SNX10 or shCONT. (**E**) Western blot showing protein levels of PDGFRβ and ubiquitin in PDGFRβ IP group or cell lysates following transduction with 1 of 2 shRNAs targeting SNX10 or shCONT following treatment with MG132. Samples were run in a single gel with entire gels shown in the supplemental material. (**F** and **G**) Normalized cell viability of (**F**) GSC CW468 and (**G**) GSC 3565 following transduction with shRNAs targeting PDGFRβ compared to shCONT over a 6-day time course. *n* = 3. Significance was determined by 2-way ANOVA with Tukey’s multiple-comparison test. (**H**) Normalized cell viability of GSC 3565 following transduction with shRNA targeting SNX10 with or without PDGFRβ overexpression compared to shCONT over a 6-day time course. *n* = 3. Significance was determined by 2-way ANOVA with Tukey’s multiple-comparison test. (**I**) Limiting dilution assay (LDA) in GSC 3565 following transduction with an shRNA construct targeting SNX10 with or without PDGFRβ overexpression compared to shCONT. (**J** and **K**) Normalized cell viability of GSC 23 following transduction with shRNAs targeting SNX10 compared to shCONT over varying concentrations of (**J**) lenvatinib or (**K**) pazopanib. Data are presented as mean ± SD. Significance was determined by 1-way ANOVA with Tukey’s multiple-comparison test (**A** and **B**) or 2-way ANOVA with Tukey’s multiple-comparison test (**G** and **H**). Significance in **I** was determined using extreme limiting dilution assays as described in Hu and Smyth (79). **P < 0.01; ***P < 0.001. NS, not significant.

Next, we explored the mechanisms underlying the regulation of SNX10 in PDGFRβ signaling, including possible roles at the pre- and posttranscriptional levels. Quantitative reverse transcriptase PCR (qRT-PCR) analysis in GSCs indicated that knockdown of SNX10 did not significantly alter the mRNA expression of PDGFA–D ligands or PDGFRβ transcripts (Figure 11, A and B). Thus, we reasoned that SNX10 regulated PDGFRβ signaling by influencing the protein level of PDGFRβ at a posttranscriptional level. Using cycloheximide treatment to arrest new protein synthesis, we observed that SNX10 knockdown accelerated protein degradation of PDGFRβ (Figure 11, C and D), suggesting a role for SNX10 in maintaining PDGFRβ stability. As protein ubiquitination is a common mechanism controlling protein degradation, we explored whether PDGFRβ ubiquitination was regulated by SNX10. PDGFRβ ubiquitination increased after SNX10 knockdown (Figure 11E). Thus, SNX10 might regulate the ubiquitination of PDGFRβ by endosomal sorting.

To interrogate the functional significance of PDGFRβ regulation by SNX10, PDGFRβ expression was knocked down with multiple shRNAs, revealing impaired GSC growth measured by both CellTiter-Glo assays and EdU incorporation assays (Figure 11, F and G, and Supplemental Figure 6, A and B), recapitulating the importance of PDGFRβ in GSC biology. In rescue experiments, PDGFRβ overexpression restored viability and stemness in SNX10-depleted GSCs (Figure 11, H and I), suggesting that SNX10 functions primarily through control of PDGFRβ. As SNX10 regulated PDGFRβ protein levels, we next tested whether SNX10 knockdown could alter cellular sensitivity to 2 PDGFRβ inhibitors, lenvatinib and pazopanib. Following SNX10 knockdown, the IC_50_ values of lenvatinib and pazopanib increased, suggesting that SNX10 expression affects resistance to these agents (Figure 11, J and K), with possible clinical relevance. Altogether, these data suggest that SNX10 promotes GSC viability and stemness through its posttranscriptional control of PDGFRβ protein stability in GSCs via endosomal protein sorting.

### SNX10 is a therapeutic target in glioblastoma and informs poor patient prognosis.

**Figure 12:**
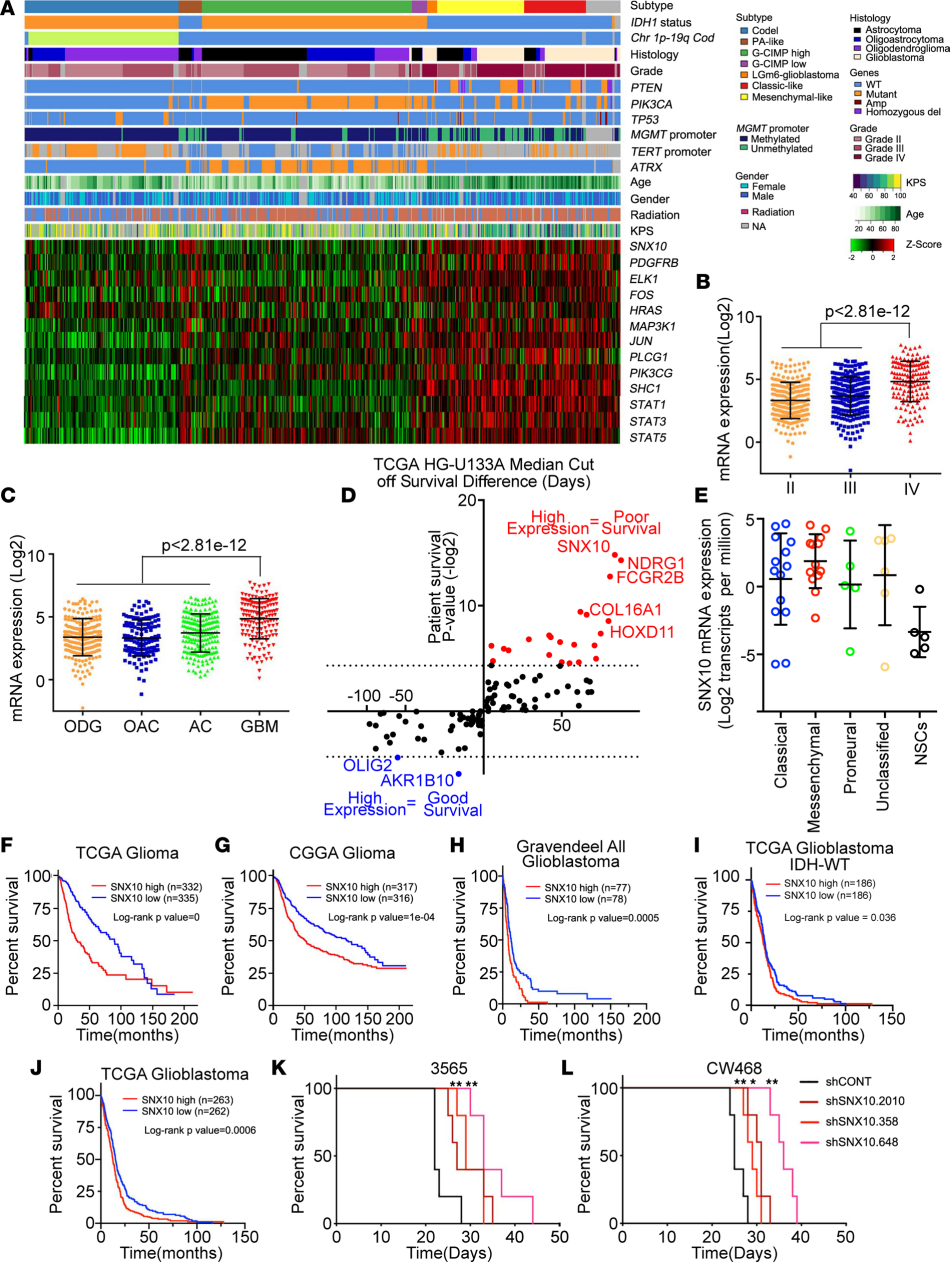
SNX10 is a useful clinical target in glioblastoma, with prognostic importance in patient data sets, and is important for in vivo tumor formation capacity. (**A**) RNA-seq, whole exome, and clinical data (667 cases) were aggregated from TCGA glioblastoma (GBM) and low-grade glioma (LGG) data sets to visualize the expression of SNX10, PDGFRβ, and PDGFRβ signature genes across glioma. Codel, co-deletion of chromosomes 1p and 19q; PA-like, pilocytic astrocytoma–like; CIMP, glioma-CpG island methylator phenotype; LGm6-GBM, a subgroup enriched for histologic low-grade gliomas but also contains a subset of tumors with GBM-defining histologic criteria; KPS, Karnofsky Performance Status. (**B** and **C**) *SNX10* mRNA levels based on (**B**) glioma grade or (**C**) glioma histology in patients from TCGA glioma data sets. ODG, oligodendroglioma; OAC, oligoastrocytoma; AC, astrocytoma; GBM, glioblastoma. (**D**) Analysis of patient survival in TCGA glioblastoma microarray (HG-U133A) data set for each gene defined in Figure 1G. The *x* axis indicates the difference in days between the high-expressing group and low-expressing group, with positive values indicating that high mRNA expression is associated with poor patient prognosis. Median expression value for each gene was used as a cutoff. The *y* axis indicates the significance of the prognostic effect. (**E**) *SNX10* mRNA expression (log_2_-transformed transcripts per million) assessed by RNA-seq in 38 GSCs and 5 NSCs used in Figure 1A stratified by transcriptional subtype (27). (**F** and **G**) Kaplan-Meier curves showing survival of all glioma patients in the (**F**) TCGA or (**G**) CGGA data sets stratified by the median *SNX10* mRNA expression. (**H**) Kaplan-Meier curve showing survival of all glioblastoma patients in Gravendeel data sets stratified by the median *SNX10* mRNA expression. (**I**) Kaplan-Meier curve showing survival of glioblastoma patients with wild-type IDH in TCGA data sets, stratified by the median *SNX10* mRNA expression. (**J**) Kaplan-Meier curve showing survival of all glioblastoma patients in TCGA data sets, stratified by the median *SNX10* mRNA expression. (**K** and **L**) Kaplan-Meier curves showing the time until onset of neurological signs in intracranial xenografts derived from (**K**) GSC 3565 or (**L**) GSC CW468 transduced with 1 of 3 independent nonoverlapping shRNAs targeting SNX10 or a nontargeting shRNA (shCONT). *P* values were calculated by 1-way ANOVA with Tukey’s multiple-comparison test (**B** and **C**) or log-rank test (**F**–**L**). *P < 0.05; **P < 0.01.

To better understand the potential clinical relevance of SNX10 in glioma patients, we interrogated clinical glioma data sets in The Cancer Genome Atlas (TCGA). *SNX10* expression strongly correlated with IDH1 status, tumor histology, and tumor grade; *SNX10* expression was higher in patients with wild-type IDH1 and glioblastoma patients (Figure 12, A–C). Consistent with the reduced PDGFRβ levels upon SNX10 knockdown in GSCs, *SNX10* expression correlated with a PDGFRβ signature score in glioma patients (Figure 12A). Among the 180 genes selected in our initial target prioritization strategy, *SNX10* was one of the top genes for which high expression was associated with poor patient prognosis in the TCGA glioblastoma data set (Figure 12D) and was upregulated in all subtypes of GSCs compared with NSCs (Figure 12E). In the clinical data sets, high mRNA expression of *SNX10* portended poor glioma patient prognosis in the TCGA and Chinese Glioma Genome Atlas (CGGA) (Figure 12, F and G). Furthermore, *SNX10* expression levels negatively correlated with prognosis of glioblastoma patients in TCGA, CGGA and Gravendeel glioblastoma data sets, including in analyses restricted to patients with wild-type IDH1 (Figure 12, H–J, and Supplemental Figure 5D). Supporting PDGFRβ regulation by SNX10, a negative correlation between SNX10 expression and glioblastoma patient prognosis was observed in the proneural transcriptional subtype, which is characterized by elevated PDGFR signatures (Supplemental Figure 6, E–G). To evaluate the proof-of-principle therapeutic potential of SNX10 in glioblastoma, we performed SNX10 knockdown in 2 patient-derived GSCs using 3 independent shRNAs and implanted cells into the brains of immunocompromised mice. Mice bearing orthotopic intracranial xenografts derived from knockdown cells displayed a prolonged time to onset of neurological signs compared with xenografts derived from cells transduced with nontargeting control (Figure 12, K and L). Taken together, these results show that SNX10 is required for glioblastoma growth in vivo and higher SNX10 expression portends worse prognosis in both glioma and glioblastoma patients, suggesting SNX10 as a potential therapeutic target with clinical utility for glioblastoma patients.

## Discussion

Dysregulated endocytosis and membrane trafficking of RTKs mediated by endosomes maintains glioblastoma malignancy (46). SNX family proteins have been implicated in endosomal sorting and endosomal homeostatic maintenance. However, we are not aware that prior studies have examined the role of SNX family protein regulation in RTK signaling via endosomal sorting in the context of glioblastoma. Through a multiomics approach followed by interrogation of functional dependencies using a loss-of-function dropout CRISPR screen, we identified *SNX10*, which encodes an endosomal protein sorting factor, as a selectively essential gene in GSCs. SNX10 belongs to a family of sorting nexins, which are defined by the presence of a phospholipid-binding motif (PX domain) that enables endosomal membrane interactions (47). The SNX family consists of 33 different subtypes in mammals and different members of the SNX family play distinct roles in intracellular trafficking, including roles in membrane trafficking, localization to and from recycling endosomes to late endosomes and towards lysosomal degradation, and both to and from the Golgi network as part of the retromer complex (47). SNX10 (along with SNX3, SNX11, SNX12, and several others) belongs to a class of SNX family members with a PX domain but lack other interaction domains present in the other 2 classes. SNX1 has been shown to reduce the levels of activated cell-surface localized EGFR, while PDGFR levels were unaffected (48).

SNX10 contains a PX domain structurally conserved as a binding domain of phosphoinositide (49). SNX10 has the simplest structure in the SNX family and maintains endosomal morphology, which is essential for the phagocytosis and digestion of pathogens, inflammatory responses, and antigen presentation by macrophages (50). SNX10 regulates endosome homeostasis, with overexpression leading to formation of giant vacuoles (51) and ciliogenesis during embryonic development through interactions with the V-ATPase complex (52). SNX10 is required for osteoclast differentiation and function (53) and missense mutations cause osteopetrosis, a heritable disorder of osteoclasts, following altered endocytosis (54, 55). SNX10 contributes to macrophage polarization, with knockdown favoring an antiinflammatory M2 state in inflammatory bowel disease (56). SNX10 controls metabolic reprogramming of macrophages in atherosclerosis by enhancing lysosomal biogenesis and lysosomal acid lipase, thus increasing free fatty acids to fuel mitochondrial fatty acid oxidation (57, 58). In contrast to our findings, SNX10 was reported to have a tumor suppressive effect in mouse inflammation-driven colorectal cancer models, with knockout leading to increased chaperone-mediated autophagy and mTOR activation (37, 59) and reduced autophagic degradation of SRC (60). The precise roles of SNX10 are likely cancer-type specific. In subsets of acute myelogenous leukemia cells, chromatin architecture is disrupted between *SNX10* and the nearby *HOXA* cluster (61), suggesting that *HOXA*-specific enhancers may be repurposed to enhance *SNX10* expression.

Endosomes serve as important platforms for RTK signaling, and we find that SNX10 supports endosomal PDGFRβ signaling in GSCs. Endosomal signaling has been previously reported for other RTKs in other model systems (22–26). We identified a specificity of SNX10 for regulation of PDGFRβ in GSCs, compared with other RTKs. PDGFRs play important roles in glioblastoma initiation and pathogenesis, with pharmacologic targeting effective against GSCs (62, 63). Unfortunately, clinical trials in glioblastoma with multitargeted kinase inhibitors against VEGFRs and PDGFRs, including tivozanib (64), pazopanib (65), or sunitinib (66), have not extended patient survival. The PDGFR inhibitor imatinib also failed to extend survival in clinical trials (67, 68), while trials with nilotinib are ongoing (ClinicalTrials.gov NCT01140568). Our results suggest that SNX10 expression may serve as a useful biomarker for response to PDGFR inhibition and may allow for stratification of patients most likely to respond. Additionally, therapeutic targeting of the endosomal signaling axis through inhibition of SNX10 or STAT3 may serve as a useful therapeutic strategy. In conclusion, SNX10 is a key GSC dependency that promotes endosomal PDGFRβ signaling via selective growth inhibition using SNX10 knockdowns in GSCs as compared to DGCs and NSCs. In addition to the plasma membrane, endosomes exist as a key platform for oncogenic signaling, revealing an additional vulnerability in glioblastoma (Supplemental Figure 7).

## Methods

### Mice and in vivo tumorigenesis.

NSG mice (NOD.Cg-*Prkdc^scid^* *Il2rg^tm1Wjl^*/SzJ, strain 005557, The Jackson Laboratory) were used to assess tumor growth by intracranially transplanting 5 × 10^4^ GSCs into the right cerebral cortex (coordinates from the bregma: *x* = 2 mm, *y* = –1 mm, *z* = –3.5 mm). All mice were monitored daily until the appearance of neurological signs or signs of morbidity, at which point the mice were sacrificed. Hunched posture, gait changes, lethargy, and weight loss were included in the neurological signs or signs of morbidity. Four- to 6-week-old male and female NSG mice were randomly selected and maintained in a specific pathogen–free facility in a 12-hour light/12-hour dark cycle at UCSD.

### Cell growth and sphere formation assay.

Cell growth was measured by CellTiter-Glo assays (Promega, G7572) in 96-well plates. Typically, 1 × 10^3^ cells were seeded per well with at least 3 replicates and then relative cell numbers were measured at desired time points by CellTiter-Glo assay based on the manufacturer’s instructions. All measurements were normalized to the data collected on day 0 and are presented as mean ± SD. GSC sphere formation capacity was measured by in vitro LDA. Briefly, different numbers of GSC cells (100, 50, 25, and 10) were plated into individual wells in 96-well plates with at least 6 replicates. The numbers of spheres in each well were counted 8 days later and the frequencies of stem cells were estimated by extreme limiting dilution analysis using software available at http://bioinf.wehi.edu.au/software/elda, as previously described.

### GSCs, neural stem cells, and non-malignant brain cultures.

All GSCs were derived from human glioblastoma specimens in our lab or by other labs and passaged through intracranial or subcutaneous mouse xenografts. See Tables 1 and 2 for more information. GSCs are functionally defined by their capacity to continuously self-renew in serial xenograft transplantation assays. GSCs used in this study were extensively functionally characterized using in vitro and in vivo LDAs and serial tumor formation assays. Nonmalignant brain cultures were derived from human epilepsy resection specimens in our lab. HNP1 (human neural progenitors), derived from human embryonic stem cells, were obtained from ArunA Biomedical (hNP7013.1). ENSA (neural progenitor cells), derived from human embryonic stem cells, were obtained from Millipore (SCR055). NSC11 are human iPSC-derived neuronal stem cells and were obtained from ALSTEM (hNSC11). All GSCs and neuronal stem cells cultured in Neurobasal medium (Invitrogen, 12348017) supplemented with B27 (Invitrogen, 17504044), EGF (20 ng/ml; R&D Systems, 236-EG), bFGF (20 ng/ml; R&D Systems, 233-FB), 1% L-GlutaMAX (Invitrogen, 35050061), 1% sodium pyruvate (Invitrogen, 11360070), and 1% penicillin/streptomycin (Invitrogen, 15070063). Nonmalignant brain cultures were cultured in mixed medium containing 50% Neurobasal medium (Neurobasal medium plus B27, EGF, bFGF, 1% L-glutamine, 1% sodium pyruvate, and 1% penicillin/streptomycin) and 50% Dulbecco’s modified Eagle’s medium (DMEM) (DMEM plus 10% FBS, 1% L-glutamine, 1% sodium pyruvate, and 1% penicillin/streptomycin). For generation of DGCs, GSCs were cultured in DMEM supplemented with 10% FBS (Sigma-Aldrich) for 1 week to induce differentiation. Short tandem repeat analyses were performed to authenticate the identity of each cell model used in this study at least once a year. Mycoplasma in cellular supernatant was tested at least twice a year by qPCR.

### Apoptosis assays.

Apoptosis was assessed using the FITC–Annexin V Apoptosis Detection Kit from BD Biosciences (catalog 556547) according to the manufacturer’s instructions. Samples were analyzed using flow cytometry on a BD LSR Fortessa flow cytometer. In brief, FSC and SSC were used to gate glioma cells and exclude cell debris. Negative, PI, and FITC–Annexin V single staining samples were used to define the negative population.

### Immunofluorescent staining.

Immunofluorescent staining was performed with cells plated on coverslips (Electron Microscopy Sciences) treated with Matrigel (Corning, 354227). Cells were fixed with 4% paraformaldehyde, followed by permeabilization with 0.1% PBST and blocking with 5% normal goat serum in 0.1% PBST, 15 minutes at room temperature. Cells were incubated with anti–cleaved caspase 3 (Cell Signaling Technology, 9664; 1:500) antibodies overnight at 4°C. Then, the cells were rinsed 3 times with PBS, 5 minutes each, followed by incubation with secondary antibodies conjugated with desired dyes (Invitrogen, 1:500) supplemented with 1 μg/mL DAPI for 2 hours at room temperature. Slides were then washed with PBS and mounted with mounting medium (Life Technologies) and processed for imaging by confocal microscopy.

### RNA isolation and qPCR.

Total RNA was isolated with TRIzol reagent (Invitrogen, 15596018) and cDNA was synthesized using a High Capacity cDNA Reverse Transcription kit (Life Technologies). Quantitative real-time PCR was performed to measure the relative expression of specific mRNAs using an Applied Biosystems 7900HT cycler or Bio-Rad CFX 9600 with SYBR Green PCR Master Mix (Life Technologies, A25778). qPCR primers are listed in Table 3.

### Plasmids and lentiviral transduction.

Lentiviral clones expressing nonoverlapping shRNAs targeting human SNX10 (TRCN0000134817, TRCN0000135959, TRCN0000134391) or a nontargeting control shRNA were obtained from Sigma-Aldrich. sgRNAs targeting human SNX10 were designed with the sgRNA design tool in the GPP Web Portal (https://portals.broadinstitute.org/gpp/public/seq/search; Broad Institute). Annealed oligos after T4 polynucleotide kinase treatment were then inserted into the LentiCRISPRv2 backbone (Addgene, 52961). 293FT cells were transfected with the transfer plasmids together with the packaging vectors pCMV-dR8.2 dvpr (Addgene, 8455) and pCI-VSVG (Addgene, 8454) using a standard calcium phosphate transfection method in DMEM plus 10% FBS. Twelve hours after transfection, culture medium was changed to complete Neurobasal medium. For shRNAs, medium containing virus particles was collected and centrifuged at 500*g* for 10 minutes at 4°C. The supernatant was filtered through a 0.45 μm filter for immediate use or stored at –80°C. See Table 4 for shRNA plasmid information.

Previously published CRISPR screening data were derived from Macleod et al. (34).

### Western blotting.

Cells were collected and suspended in RIPA buffer supplemented with protease inhibitors and phosphatase inhibitor. The suspended cells were kept on ice for 30 minutes. The lysates were centrifuged at 20,000*g* for 10 minutes at 4°C and the supernatant was collected and processed for protein concentration quantification by Bradford assay. The protein samples were processed for immediate use by mixing with SDS Laemmli loading buffer and boiling for 10 minutes. Equal amounts of protein were used for electrophoresis with PAGE gels. The PVDF membranes with proteins were blocked with TBST supplemented with 3.5% BSA at room temperature for 30 minutes, followed by incubation with primary antibodies overnight at 4°C. Primary antibodies are listed in Table 5. After rinsing with TBST, the PVDF membranes were incubated with secondary antibodies, followed by rinsing with TBST and signal development using SuperSignal West Pico PLUS Chemiluminescent Substrate (Thermo Fisher Scientific, 34580) and capture on Autoradiography Film (Denville Scientific). The developed films were scanned with an Epson Perfection V600 Photo.

### In vitro CRISPR screening and data analysis.

A custom CRISPR/Cas9 sgRNA library was designed to target 180 genes with 5 sgRNAs targeting each gene and 100 nontargeting sgRNAs (1,000-sgRNA library) in the LentiCrisprV2 vector (Addgene, 52961). The library was stably transduced into GSCs by lentiviral infection with a multiplicity of infection (MOI) of approximately 0.3 and coverage of 500. Cells were selected with puromycin for 48 hours and then propagated in standard GSC cell culture conditions for 7 days. Genomic DNA was extracted from GSCs and sequencing libraries were generated by PCR amplification of the inserted sgRNA sequences and addition of sequencing adapters. Sequencing was performed at the UCSD genomics core. Sequencing quality control was performed using FASTQC and sgRNA dropout was calculated using the MAGeCK-VISPR pipeline using the MAGeCK-MLE algorithm (29). Gene overlaps were calculated and visualized using an Upset plot. Oligonucleotide sequences are listed in Table 6.

### RNA-seq analysis.

RNA-seq data (GEO GSE119834) were obtained from a previously published report by Mack et al. (27). Transcript quantification was performed using Salmon in the quasi-mapping mode (69). Salmon “quant” files were converted using Tximport (https://bioconductor.org/packages/release/bioc/html/tximport.html) and differential expression analysis was performed using DESeq2 (70). Gene set enrichment analysis (GSEA) was performed by selecting differentially expressed genes (FDR-corrected *P* value < 0.001, log_2_[fold change] > 2), generating a preranked list, and inputting the preranked list into the GSEA desktop application (http://software.broadinstitute.org/gsea/ downloads.jsp) (71, 72). Pathway enrichment bubble plots were generated using the Bader Lab Enrichment Map Application and Cytoscape (http://www.cytoscape.org) (73).

For SNX10 knockdown experiments, TRIzol reagent (Sigma-Aldrich) was used to isolate total cellular RNA according to the manufacturer’s instructions. Three GSCs were utilized with a nontargeting shRNA control and 3 independent nonoverlapping shRNAs targeting SNX10. Biological duplicates were utilized for each GSC. RNA was purified using an RNeasy kit (Qiagen) and sequenced by Novogene Corporation. FASTQ sequencing reads were trimmed using Trim Galore (https://www.bioinformatics.babraham.ac.uk/projects/trim_galore/) and transcript quantification performed using Salmon as described above. GSEA was performed by selecting differentially expressed genes (FDR-corrected *P* value < 0.01, log_2_[fold change] > 0.5 or < –0.5), generating a preranked list, and inputting the preranked list into the GSEA desktop application (http://software.broadinstitute org/gsea/downloads.jsp). Pathway enrichment bubble plots were generated using the Bader Lab Enrichment Map Application and Cytoscape (http://www.cytoscape.org). All raw and selected processed data from RNA sequencing experiments are publicly available in the NCBI Gene Expression Omnibus database (GEO GSE221714).

### ChIP-seq analysis and enhancer identification.

H3K27ac ChIP-seq data were obtained from a previously published report by Mack et al. (27) (GEO GSE119755). Single-end H3K27ac and input ChIP-seq reads were trimmed using Trim Galore and cutadapt. Reads were aligned to the hg19 human genome with BWA-MEM (Heng Li, arXiv: 1303.3997; https://arxiv.org/abs/1303.3997). BAM files were processed using SAMtools (74) and PCR duplicates removed with PicardTools (http://broadinstitute.github.io/picard/). H3K27ac peaks were called using MACS2 (75) using a ChIP input file as a control with a *P*-value cutoff of 1 × 10^–9^. BIGWIG track coverage files were generated from BAM files using the DeepTools bamCoverage command (76) and visualized in IGV (77, 78). Differential H3K27ac peaks were calculated using the DESeq2 algorithm in the DiffBind package. H3K27ac peaks were considered GSC specific for peaks with an FDR-corrected *q* value of less than 0.001 and a fold change greater than 2. Peaks were mapped to the nearest unique gene less than 20 kb from the peak. GSEA was performed by selecting differentially expressed genes (FDR-corrected *q* value < 0.001, fold change > 2), generating a preranked list, and inputting the preranked list into the GSEA desktop application (http://software.broadinstitute.org/gsea/downloads.jsp). Pathway enrichment bubble plots were generated using the Bader Lab Enrichment Map Application and Cytoscape (http://www.cytoscape.org).

Motifs were called from GSC-specific enhancer regions within glioblastoma-specific super-enhancers using the HOMER “findmotifsgenome.pl” script using the hg19 genome. The top scoring de novo motifs are presented.

### Patient database bioinformatics.

The GlioVis data portal (http://gliovis.bioinfo.cnio.es/) was used to interrogate the clinical relevance of patients with low-grade glioma or glioblastoma from TCGA, CGGA, or Gravendeel glioblastoma data sets. The survival of patients in each group was analyzed using the Kaplan-Meier method via log-rank test.

### Quantification and statistics.

One-way ANOVA was employed to assess the statistical significance in data sets having more than 2 groups. Two-way ANOVA was used to determine the statistical significance in data sets having subgroups in each main group. In LDA-based sphere formation experiments, the χ^2^ test was used for pair-wise differences in assessing the frequencies of stem populations. GraphPad Prism 6 software was used to generate Kaplan-Meier survival curves in xenograft experiments and log-rank tests were used to assess statistical significance between different groups. All experiments were performed at least 3 times independently. All data are presented as mean ± SD. A *P* value of less than 0.05 was considered significant, with *P < 0.05, **P < 0.01, ***P < 0.001. No statistical methods or criteria were used to estimate sample size or to include or exclude samples. The investigators were not blinded to the group allocation during the experiments.

### Study approval.

All animal experiments were performed under an animal protocol approved by Institutional Animal Care and Use Committee at UCSD. This work did not contain research involving humans.

## Author contributions

RCG, GZ, and JNR conceived and designed the study. RCG, GZ, DL, ZD, QW, QX, and JNR developed the methodology. RCG, GZ, DL, QW, ZD, LZ, ZQ, DD, RLK, SW, and ST acquired data (provided animals, acquired patients, provided facilities, etc.). RCG, GZ, ZQ, BCP, SW, ARM, ST, LJYK, SA, and JNR analyzed and interpreted data (statistical analysis, biostatistics, computational analysis). TH developed methodology and acquired and analyzed data. JL acquired and analyzed data. MEH acquired and analyzed data and reviewed the manuscript and figures. RCG, GZ, ZD, ZQ, BCP, SW, ST, LJYK, SA, and JNR wrote, reviewed, and/or edited the manuscript. JNR provided administrative, technical, or material support (i.e., reporting or organizing data, constructing databases). Co–first authors RCG and GZ contributed equally to the conception, methodological design, acquisition of data, and reviewing of the manuscript and figures. Both authors played important roles in each element of producing the manuscript. RCG is listed before GZ as RCG conceived of the initial experimental design and approach, performed key experiments, and performed a greater role in drafting, editing, and revising the manuscript.

## Supplementary Material

Supplemental data

## Figures and Tables

**Table 1:**
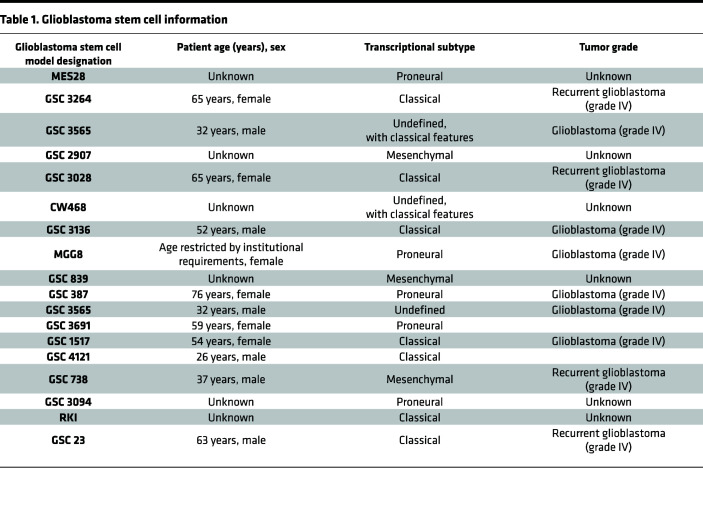
Glioblastoma stem cell information

**Table 2:**
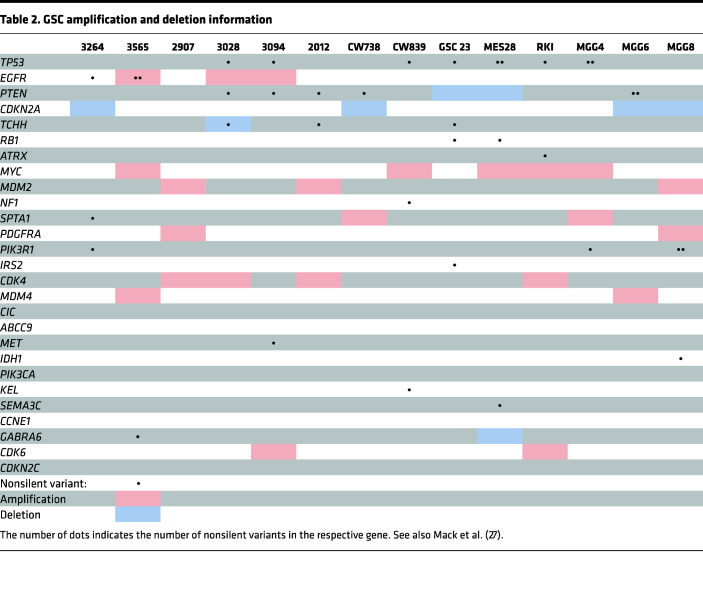
GSC amplification and deletion information

**Table 3:**
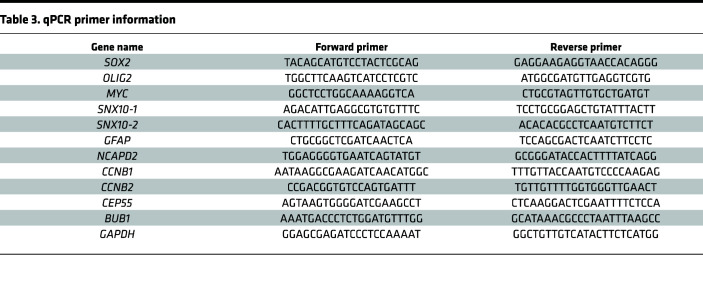
qPCR primer information

**Table 4:**
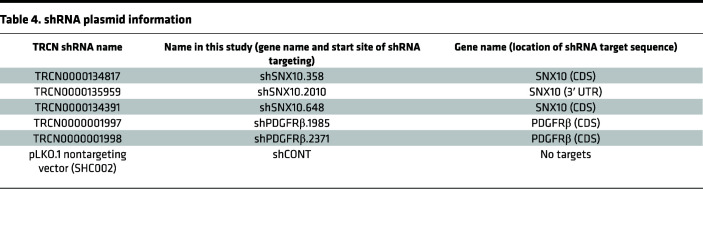
shRNA plasmid information

**Table 5:**
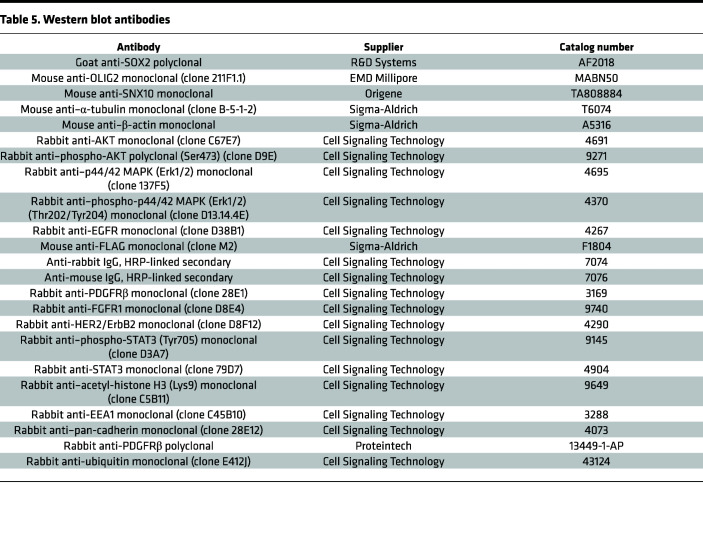
Western blot antibodies

**Table 6:**
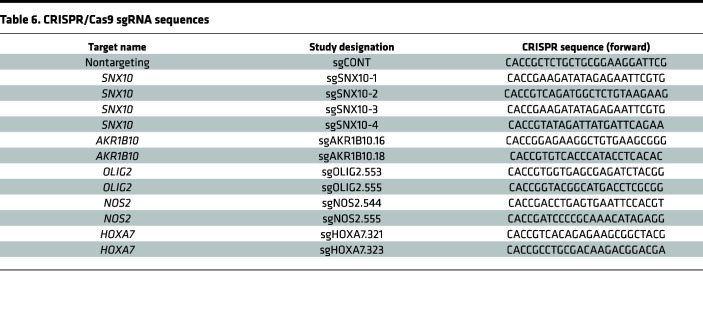
CRISPR/Cas9 sgRNA sequences

## Version 1 02/16/2023

In-Press Preview

## Version 2 03/22/2023

Electronic publication
