# Supplementary material for: Sorting nexin 10 sustains PDGF receptor signaling in glioblastoma stem cells via endosomal protein sorting
Source: JCI Insight. 2023 Mar 22;8(6):e158077. doi: 10.1172/jci.insight.158077 (PMC10070110; doi:10.1172/jci.insight.158077)
Supplement: Supplemental data [file jciinsight-8-158077-s184.pdf]

Sorting Nexin 10 sustains Platelet-Derived Growth Factor Receptor signaling in glioblastoma stem cells via endosomal protein sorting

Ryan C. Gimple[1,2,*], Guoxin Zhang[1,*], Shuai Wang[3], Tengfei Huang[3], Jina Lee[3], Suchet Taori[3], Deguan Lv[1,3], Deobrat Dixit[1,4], Matthew E. Halbert[5,6], Andrew R. Morton[2], Reilly L. Kidwell[1], Zhen Dong[7], Briana C. Prager[1,2], Leo Kim[1,2], Zhixin Qiu[1,3], Linjie Zhao[1,3], Qi Xie[1,8,9,10], Qiulian Wu[1,3], Sameer Agnihotri[3,5,6], Jeremy N. Rich[1,3,11-13,†]

[1]Division of Regenerative Medicine, Department of Medicine, University of California, La Jolla, CA 92037, USA

[2]Department of Pathology, Case Western Reserve University School of Medicine, Cleveland, OH 44106, USA

[3]UPMC Hillman Cancer Center, Pittsburgh, PA 15232, USA

[4]Sanford Burnham Prebys Medical Discovery Institute, La Jolla, CA 92037, USA

[5]Department of Neurosurgery, University of Pittsburgh Medical Center, Pittsburgh, PA, USA

[6]John G. Rangos Sr. Research Center, Children's Hospital of Pittsburgh, Pittsburgh, PA, USA

[7]La Jolla Institute for Immunology, La Jolla, CA 92037

[8]Key Laboratory of Growth Regulation and Translational Research of Zhejiang Province, School of Life Sciences, Westlake University, Hangzhou, Zhejiang, China.

[9]Westlake Laboratory of Life Sciences and Biomedicine, Hangzhou, Zhejiang, China.

[10]Institute of Basic Medical Sciences, Westlake Institute for Advanced Study, Hangzhou, Zhejiang, China

[11]Sanford Consortium for Regenerative Medicine, 2880 Torrey Pines Scenic Drive, La Jolla, CA 92037

[12]Department of Neurology, University of Pittsburgh, Pittsburgh, PA, USA

[13]Department of Neurosciences, University of California, La Jolla, CA 92037

*, These authors contributed equally.

Correspondence: Jeremy N. Rich (drjeremyrich@gmail.com)

5150 Centre Avenue, 5th Floor, Pittsburgh, PA 15232. 412-623-3364.

Key words: glioblastoma, glioblastoma stem cell, cancer stem cell, endosome, SNX10, PDGFR

Conflict of Interest Statement: The authors have declared that no conflicts of interest exist.

Supplemental Figure 1

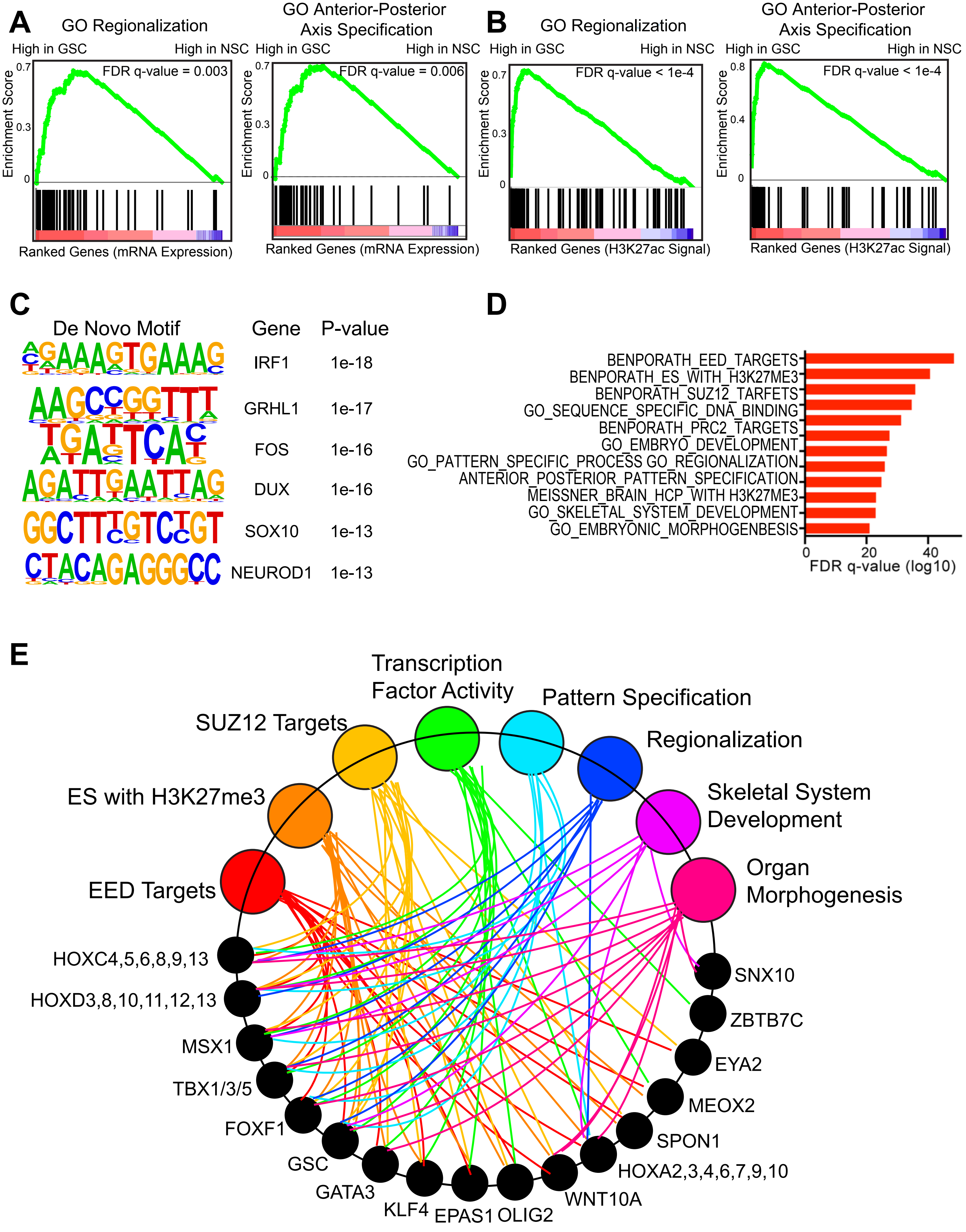

SUPPLEMENTARY FIGURE LEGENDS

Figure S1: Gene set enrichment analysis of targets identified through combinatorial epigenetic and transcriptional profiling

A. Gene set enrichment analysis (GSEA) plots of selected pathways upregulated in GSCs vs. NSCs by RNA-sequencing. FDR q-value was calculated for statistical analysis.

B. Gene set enrichment analysis (GSEA) plots of selected pathways upregulated in GSCs vs. NSCs by ChIP-sequencing. FDR q-value was calculated for statistical analysis.

C. HOMER de novo motif analysis of GSC-specific H3K27ac peaks.

D. Gene set enrichment analysis (GSEA) of 180 overlapping candidate genes identified in Figure 1G.

E. Gene set enrichment connectivity diagram showing top gene set enrichments and individual component genes of 180 overlapping candidate genes identified in Figure 1G.

Supplemental Figure 2

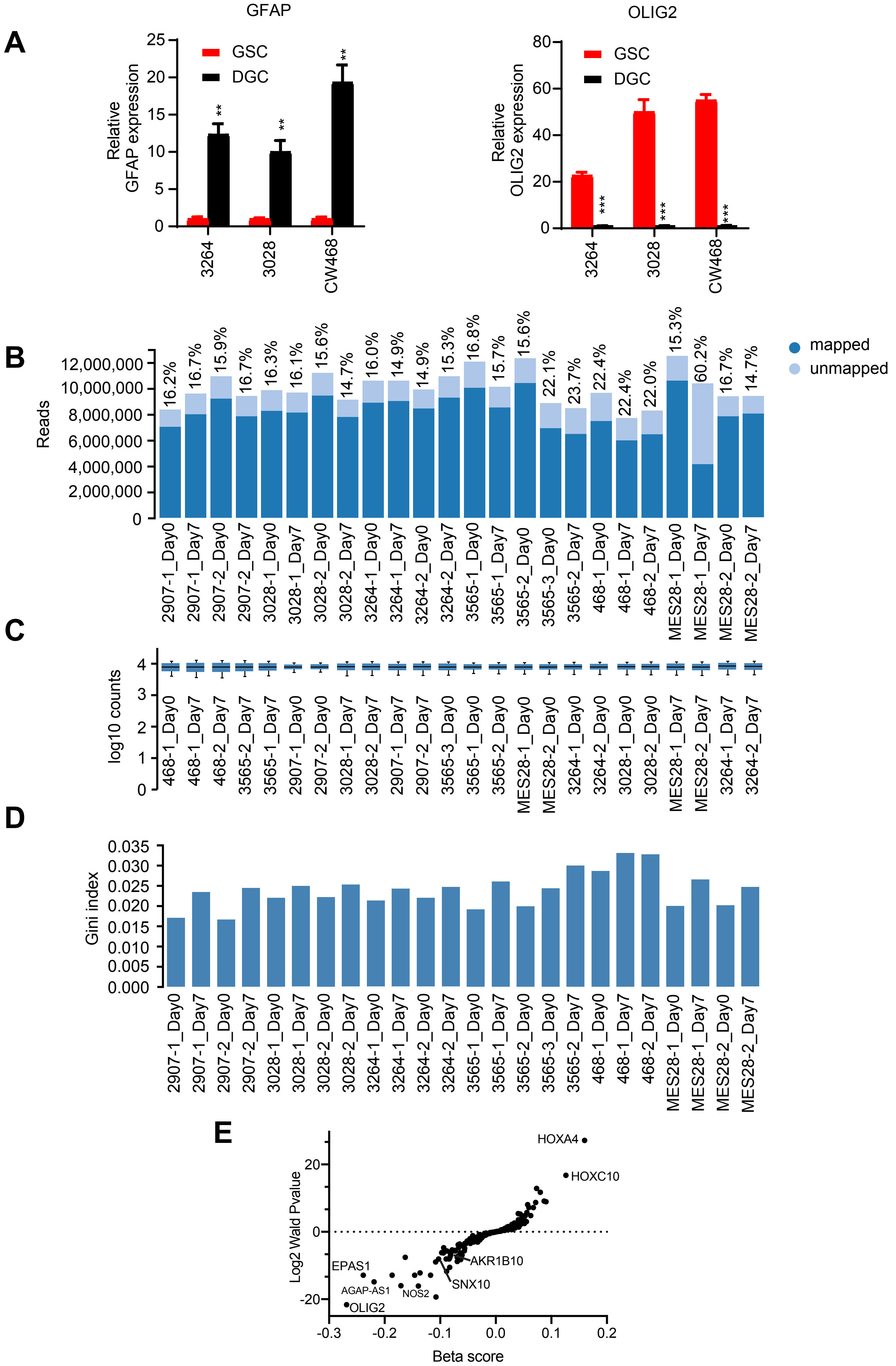

Figure S2: Targeted CRISPR screen quality control metrics.

A. q-RTPCR analysis of mRNA expression of GFAP and OLIG2 in paired GSCs and DGCs. **, p<0.01; ***, p<0.001.

B. Total number of reads from each CRISPR screening sample with percentage of mapped and unmapped reads.

C. Log10 of sgRNA counts from each CRISPR screening sample.

D. Gini index from each CRISPR screening sample.

E. Combined analysis of CRISPR screening data in all six GSCs. Negative beta-value indicates essentiality and log2 transformed wald p-value indicates significance.

Supplemental Figure 3

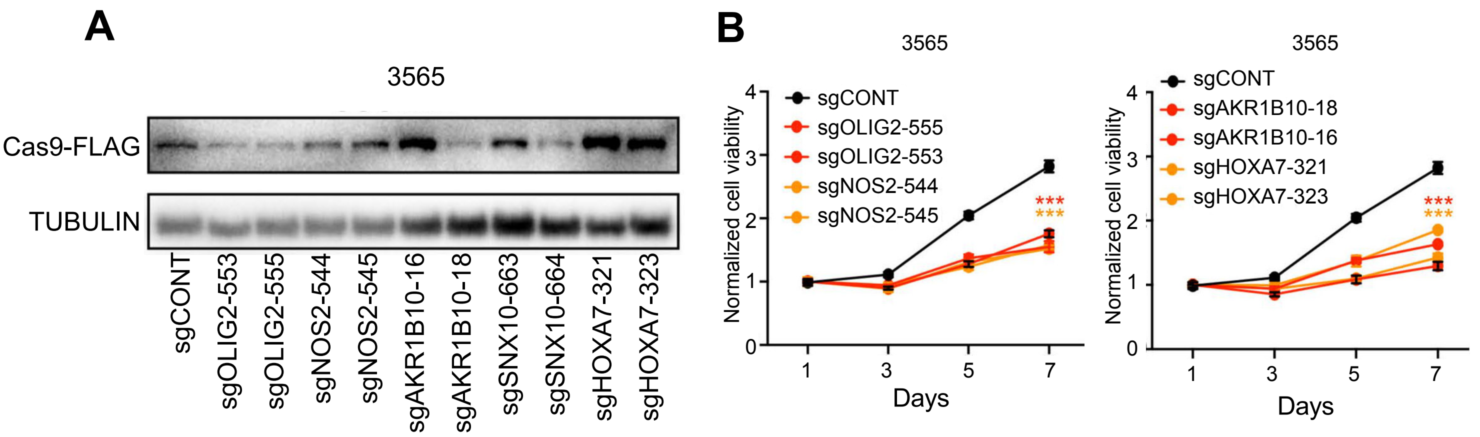

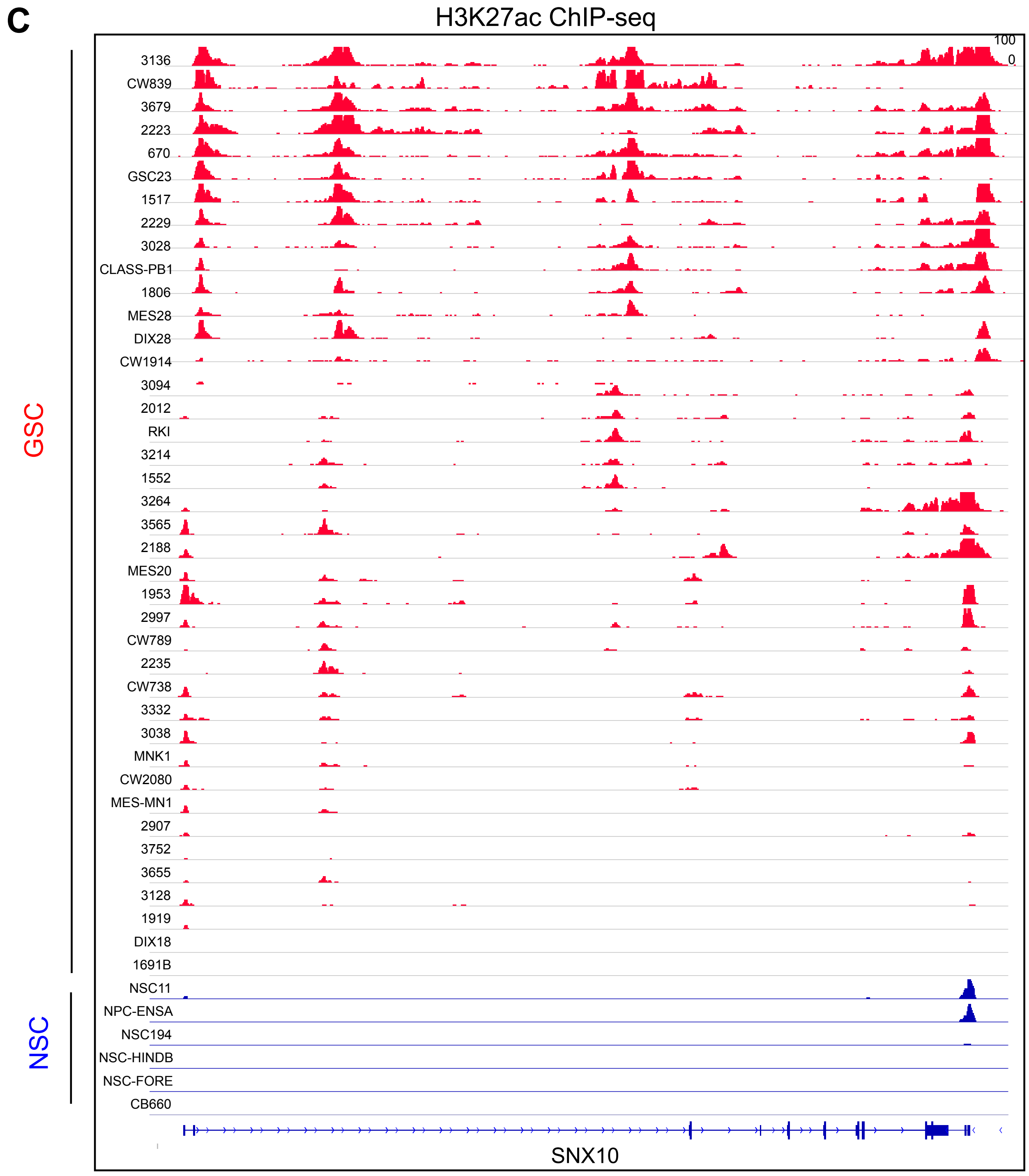

Figure S3: Validation of targeted CRISPR screening targets and SNX10 expression

A. Western blot showing transduction of the FLAG-tagged CRISPR-Cas9 construct in GSC3565 for individual sgRNA validation experiments.

B. Normalized cell viability of GSC 3565 following transduction with sgRNAs targeting OLIG2, NOS2, AKR1B10 or HOXA7 or a non-targeting sgRNA (sgCONT) over a 7-day time course. Repeated measures two-way ANOVA with Dunnett multiple test correction was used for statistical analysis.

C. Overlay of H3K27ac signal at the SNX10 locus across an overlay of 38 GSC and 5 NSCs.

Supplemental Figure 4

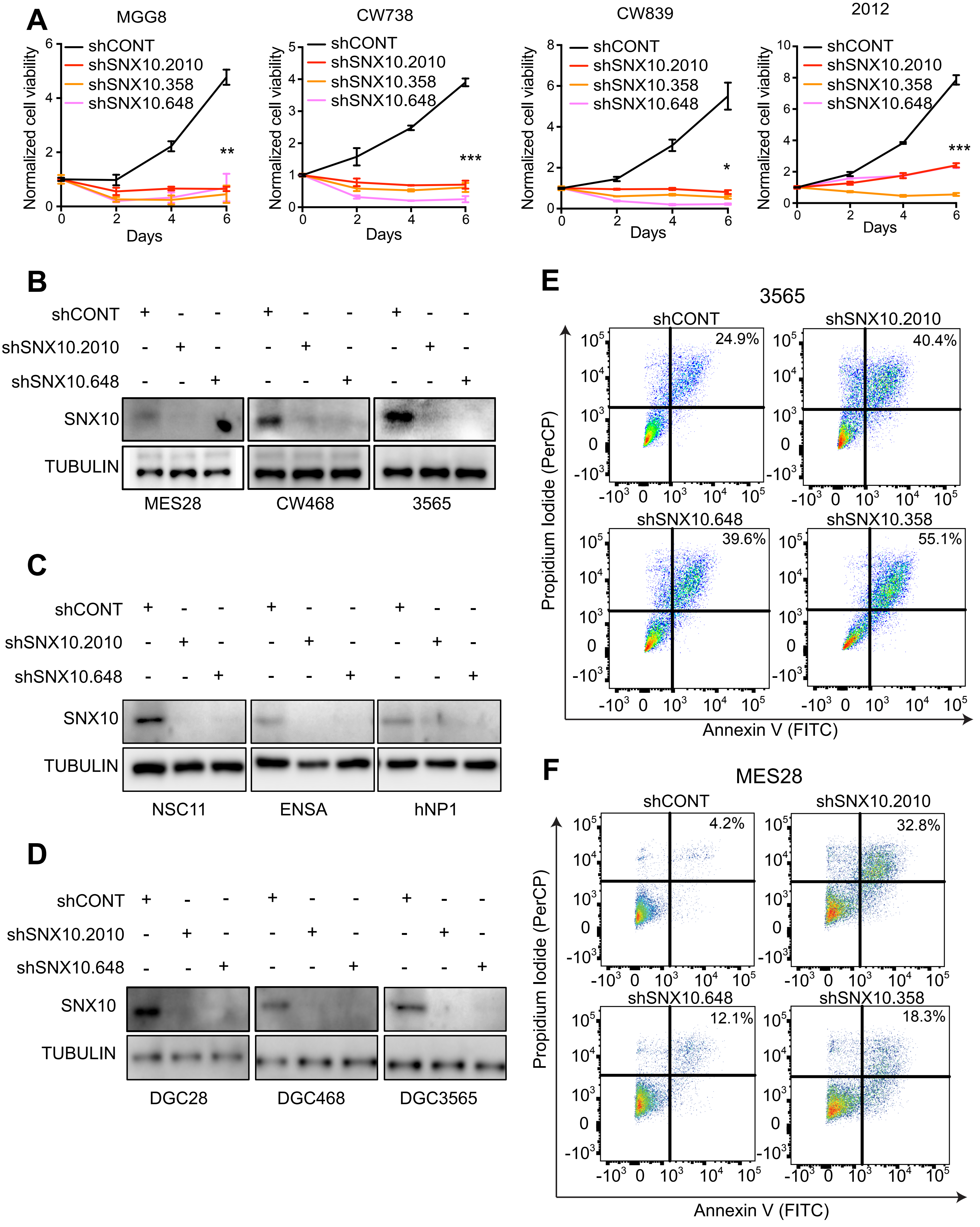

Figure S4: SNX10 knockdown reduced cell proliferation in GSCs and contributed to apoptotic cell death.

A. Normalized cell viability of GSC MGG8, GSC CW738, GSC CW839, and GSC 2012 following transduction with one of three shRNAs targeting SNX10 compared to a nontargeting shRNA (shCONT) over a 6 day time course. N=3. Data are presented as mean ± SD. Significance was determined by two-way ANOVA with Tukey's multiple comparisons. *, $p < 0.05$; **, $p < 0.01$; ***, $p<0.001$.

B. Western blot showing SNX10 protein levels in MES28, CW468 and 3565 following knockdown of SNX10 with two independent shRNAs. Tubulin was used as a loading control.

C. Western blot showing SNX10 protein levels in NSC11, ENSA, and hNP1 following knockdown of SNX10 with two independent shRNAs. Tubulin was used as a loading control.

D. Western blot showing SNX10 protein levels in DGC MES28, DGC CW468, and DGC 3565 following knockdown of SNX10 with two independent shRNAs. Tubulin was used as a loading control.

E. Flow cytometry of Annexin V/ Propidium iodide (PI) stained GSC 3565 transduced with shCONT or one of three shRNAs targeting SNX10.

F. Flow cytometry of Annexin V/ Propidium iodide (PI) stained GSC MES28 transduced with shCONT or one of three shRNAs targeting SNX10.

Supplemental Figure 5

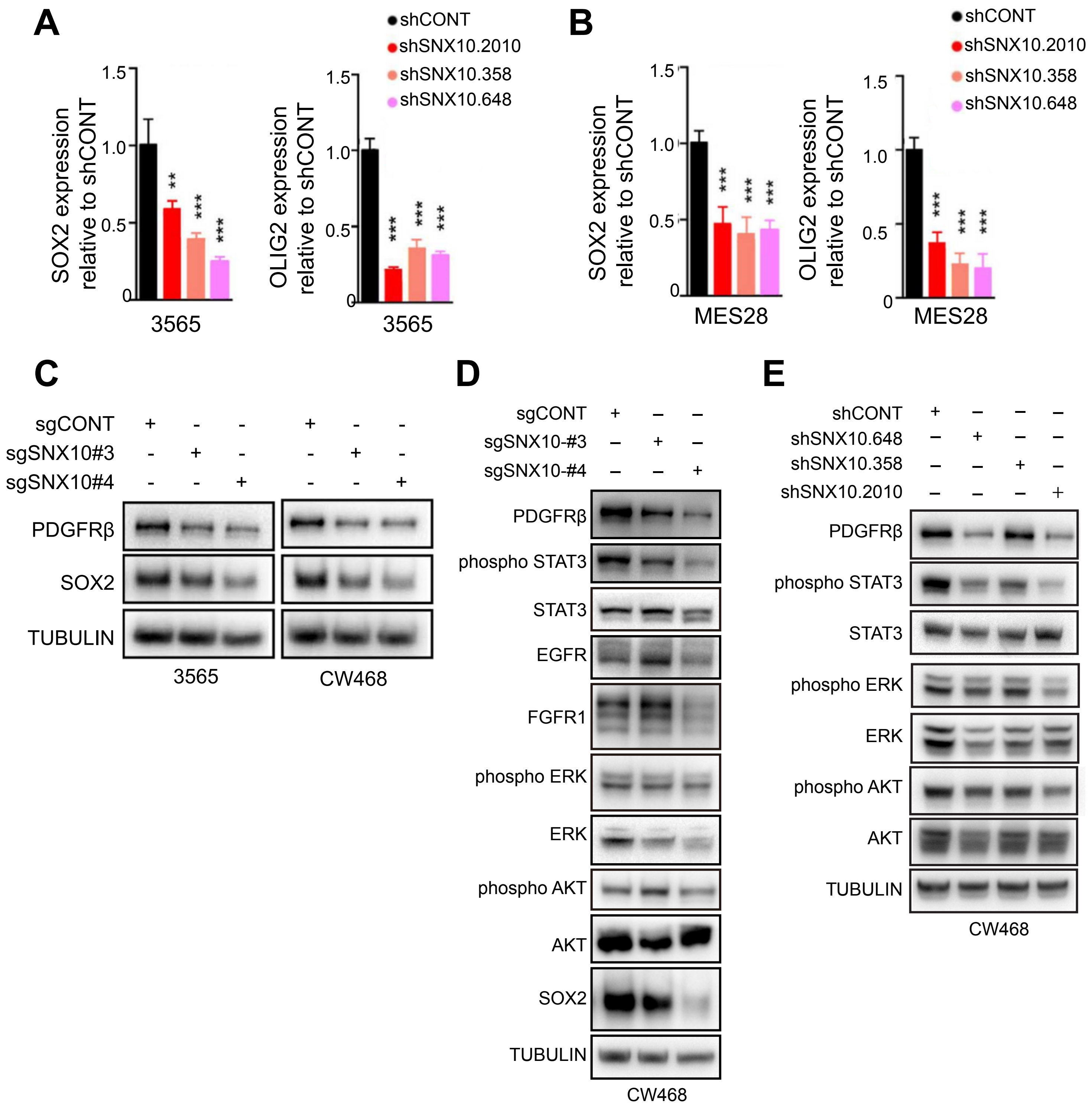

Figure S5: SNX10 is important for maintenance of GSC stemness pathways and for endosomal PDGFRβ signaling

A. mRNA expression of SOX2 (left) and OLIG2 (right) in GSC 3565 measured by qPCR following transduction with three independent non-overlapping shRNAs targeting SNX10 or a non-targeting control shRNA (shCONT). N=3. Data are presented as mean ± SD. Significance was determined by one-way ANOVA with Tukey's multiple comparisons. ** $p<0.01$, *** $p<0.001$.

B. mRNA expression of SOX2 (left) and OLIG2 (right) in GSC MES28 measured by qPCR following transduction with three independent non-overlapping shRNAs targeting SNX10 or a non-targeting control shRNA (shCONT). N=3. Data are presented as mean ± SD. Significance was determined by one-way ANOVA with Tukey's multiple comparisons. ** $p<0.01$, *** $p<0.001$.

C. Western blot of PDGFRβ and SOX2 in GSC 3565 or GSC CW468 following transduction with different sgRNAs targeting SNX10 compared to a non-targeting sgRNA (sgCONT). TUBULIN was used as loading control.

D. Western blot showing protein levels of selected receptor tyrosine kinases and downstream signaling factors in GSC CW468 following transduction with one of two sgRNAs targeting SNX10 or a non-targeting sgRNA (sgCONT). Tubulin was used as a loading control. Samples were run contemporaneously on separate gels with individual loading controls shown in Supplementary information.

E. Western blot showing protein levels of selected receptor tyrosine kinases and downstream signaling factors in GSC CW468 following transduction with one of three shRNAs targeting SNX10 or a non-targeting shRNA (shCONT). Tubulin was used as a loading control. Samples were run contemporaneously on separate gels with individual loading controls shown in Supplementary information.

Supplemental Figure 6

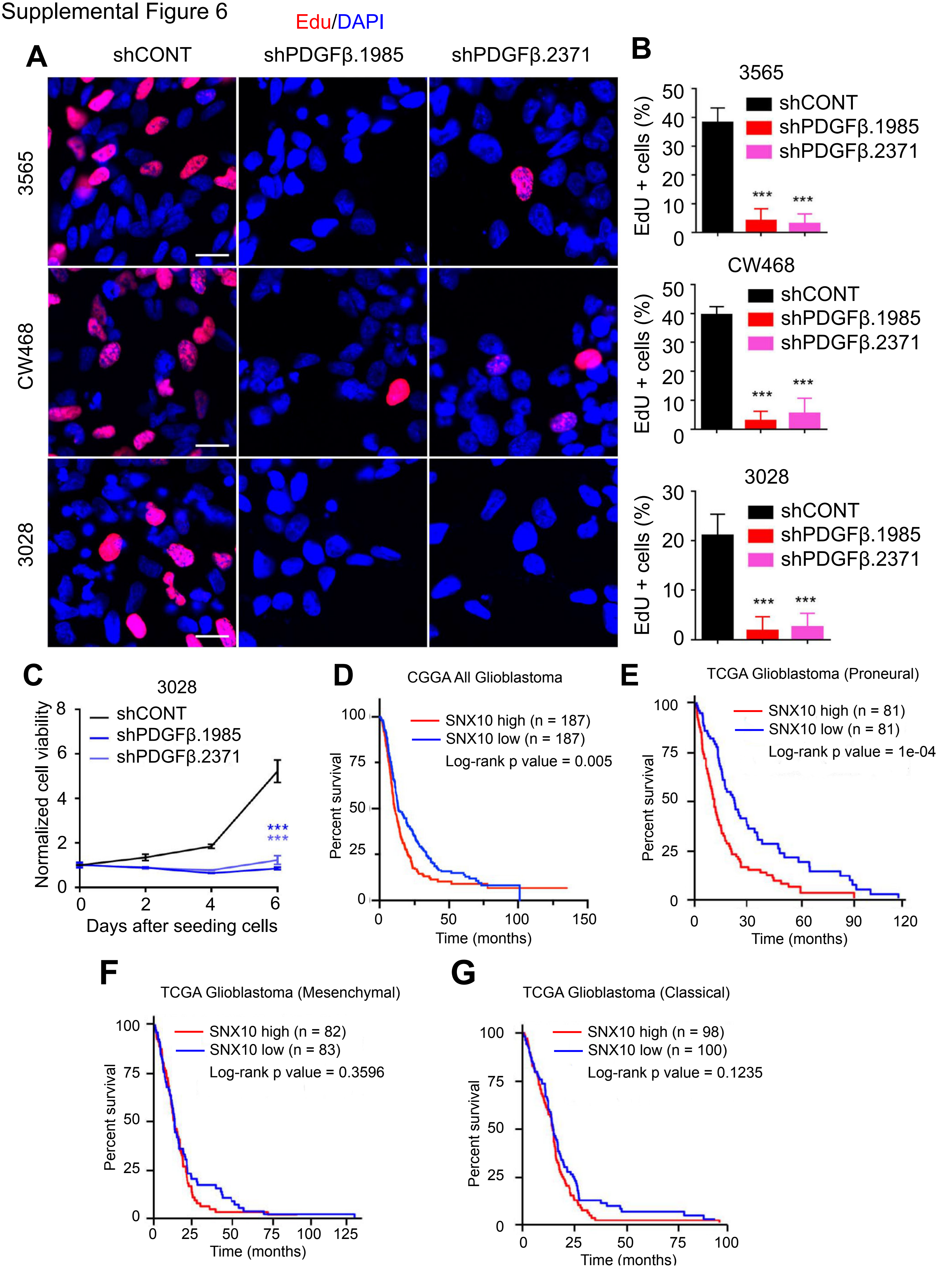

Figure S6: SNX10 portends poor prognosis in proneural subtype of glioblastoma patients.

A. Immunofluorescence images of EdU positive cells in different GSCs transduced with shCONT or one of two shRNAs targeting PDGFRβ. EdU is in red, DAPI in blue. Scale bars represent 20μm.

B. Quantification of EdU positive cells in different GSCs transduced with shCONT or one of two shRNAs targeting PDGFRβ. N = 3. Data are presented as mean ± SD. Significance was determined by one-way ANOVA with Tukey's multiple comparisons. ***, $p<0.001$.

C. Normalized cell viability of GSC 3028 following transduction with sgRNAs targeting SNX10 compared to a non-targeting sgRNA (sgCONT) over a 6-day time course. N = 3. Data are presented as mean ± SD. Significance was determined by two-way ANOVA with Tukey's multiple comparisons. ***, $p<0.001$.

D. Kaplan-Meier curve showing survival of all glioblastoma patients in the CGGA datasets stratified by the median mRNA expression of SNX10. Log-rank analysis was used for statistical analysis.

E. Kaplan-Meier curve showing survival of glioblastoma patients of proneural subtype in TCGA datasets stratified by the median mRNA expression of SNX10. Log-rank analysis was used for statistical analysis.

F. Kaplan-Meier curve showing survival of glioblastoma patients of mesenchymal subtype in TCGA datasets stratified by the median mRNA expression of SNX10. Log-rank analysis was used for statistical analysis.

G. Kaplan-Meier curve showing survival of glioblastoma patients of classical subtype in TCGA datasets stratified by the median mRNA expression of SNX10. Log-rank analysis was used for statistical analysis.

Supplemental Figure 7

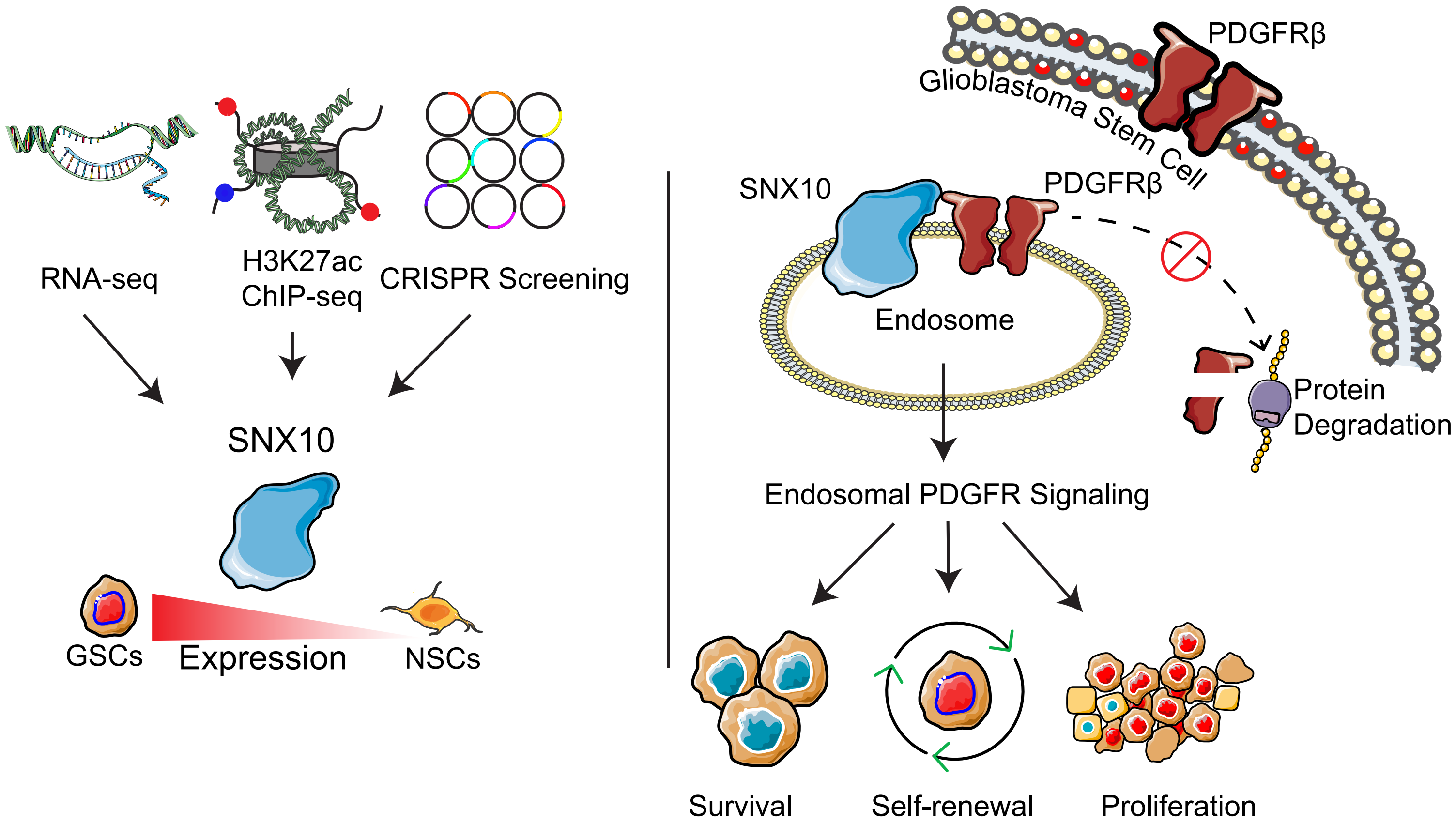

Figure S7: Model figure of target identification approach and mechanism of action

(**Left**) Model figure of target identification approach through combinatorial epigenetic and transcriptional profiling followed by a CRISPR-Cas9 loss-of-function dropout screen identifies SNX10. (**Right**) Model figure of mechanism of action of SNX10. SNX10 functions to maintain PDGF receptor signaling through control of endosomes to maintain glioblastoma stem cell proliferation and self-renewal properties.

Full unedited gel for Figure 3D

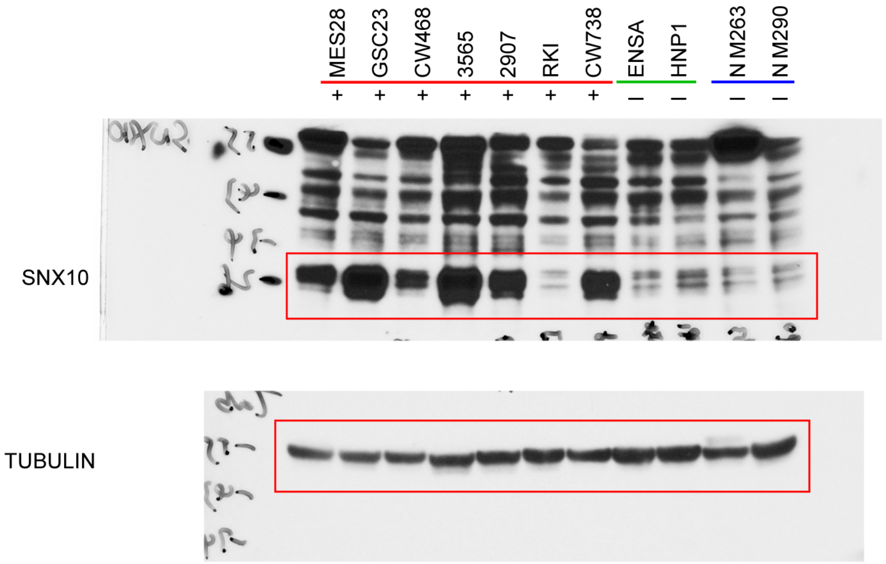

Full unedited gel for Figure 3E

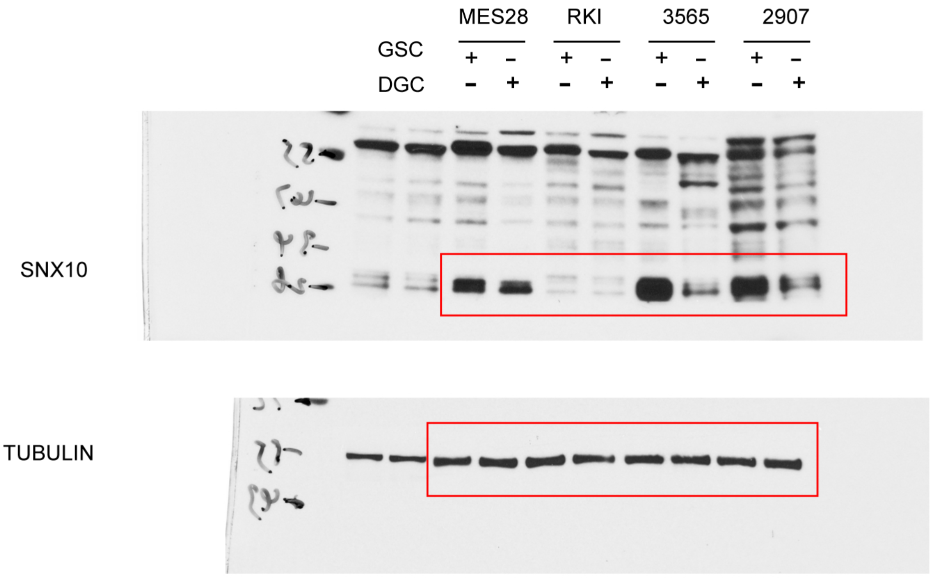

SNX10 Monoclonal Antibody (OTI3F1) Origene Cat # TA808884

Mouse monoclonal antibody to alpha-Tubulin
(clone B-5-1-2) Sigma Aldrich, Cat T6074

Full unedited gel for Figure 7C

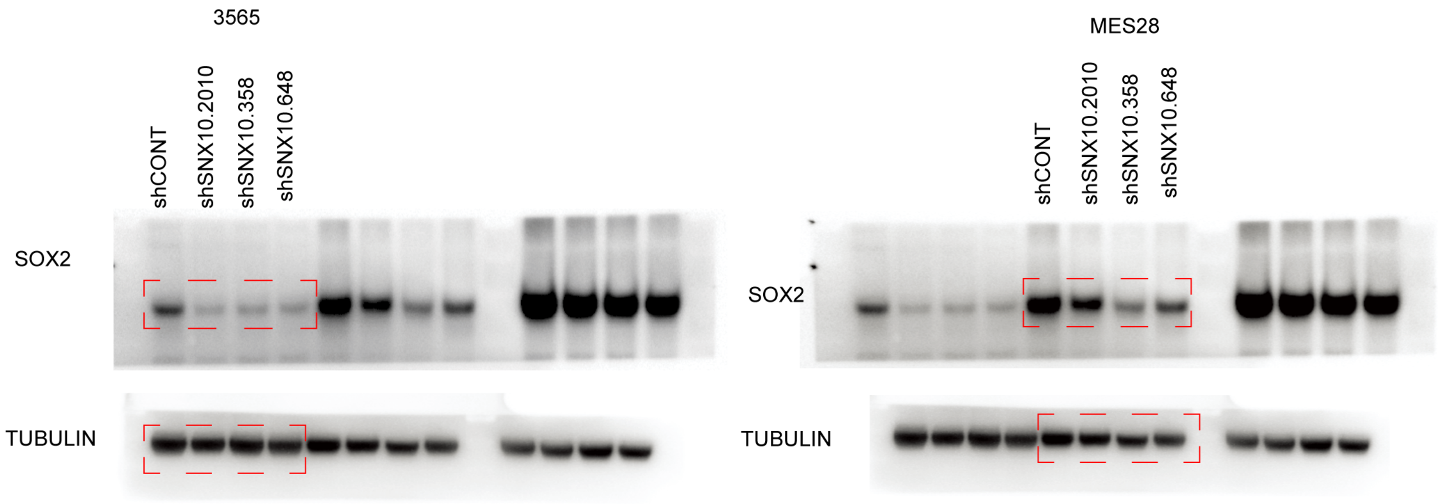

Goat polyclonal antibody to SOX2 R&D Systems Cat # AF2018

Mouse monoclonal antibody to alpha-Tubulin
(clone B-5-1-2) Sigma Aldrich, Cat T6074

Full unedited gel for Figure 8D

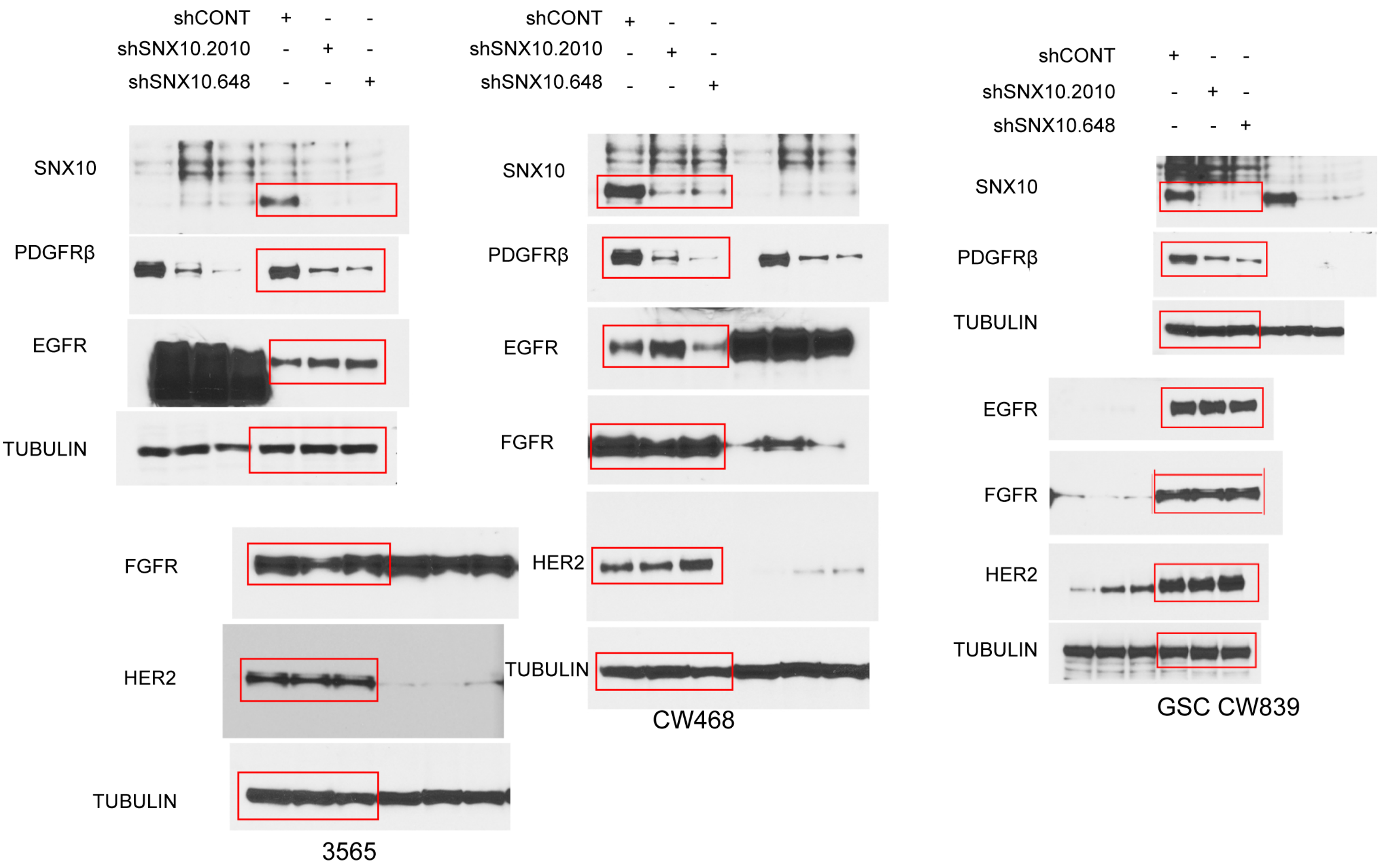

Full unedited gel for Figure 8E

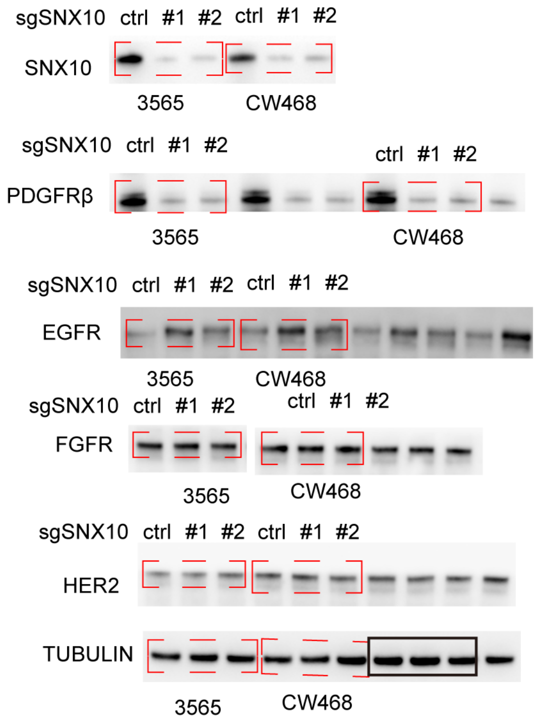

SNX10 Monoclonal Antibody (OTI3F1) Origene Cat # TA808884

PDGF Receptor β (28E1) Rabbit mAb CST #3169

Rabbit monoclonal antibody to EGF Receptor
(D38B1) CST Cat # 4267

FGF Receptor 1 (D8E4) XP® Rabbit mAb
#9740 CST Cat #9740

Mouse monoclonal antibody to alpha-Tubulin
(clone B-5-1-2) Sigma Aldrich, Cat T6074

HER2/ErbB2 (D8F12) XP® Rabbit mAb #4290

Full unedited gel for Figure 8F

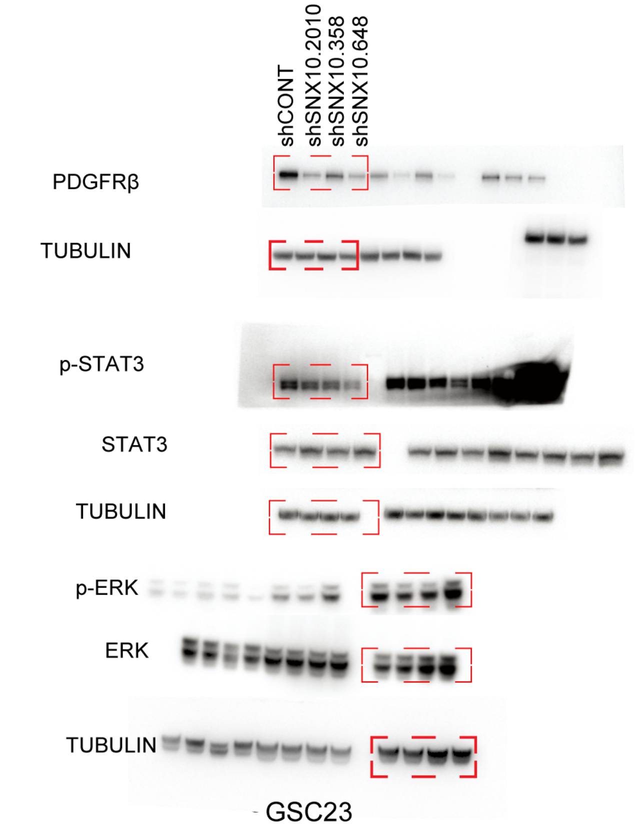

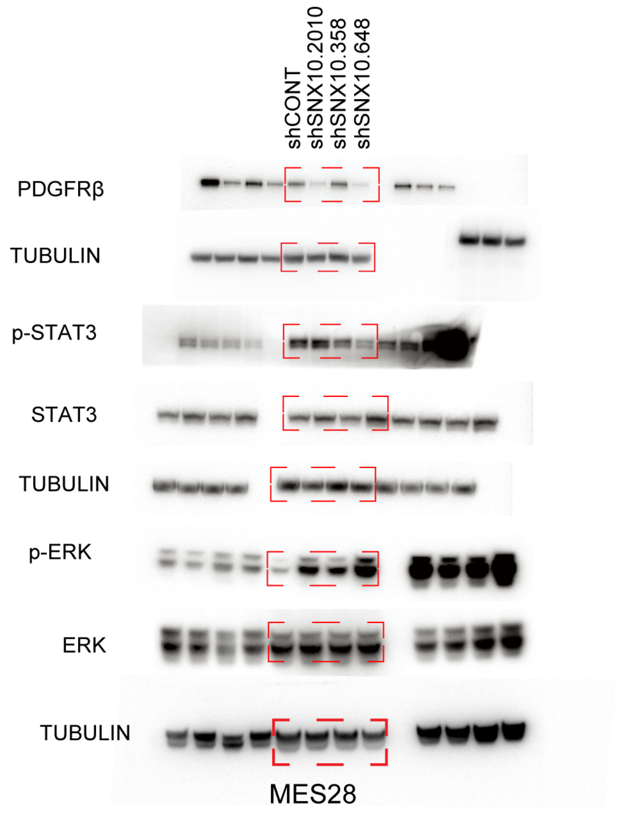

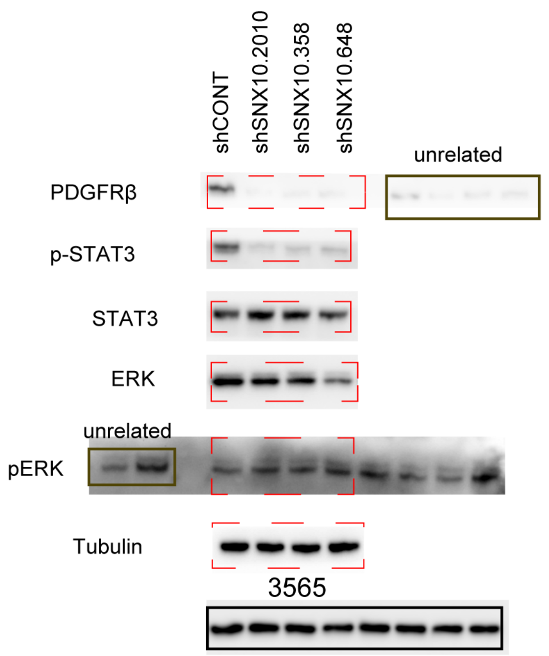

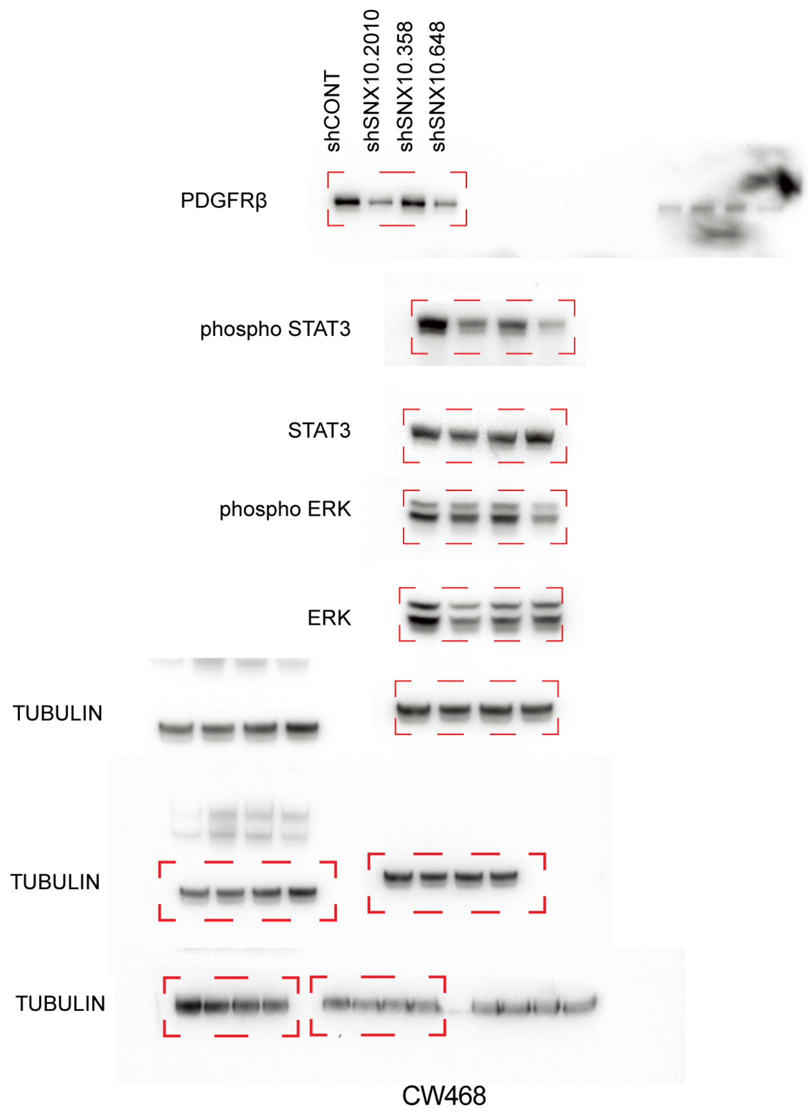

PDGF Receptor β (28E1) Rabbit mAb CST #3169

Phospho-Stat3 (Tyr705) (D3A7) XP® Rabbit mAb CST#9145

STAT3(79D7) Rabbit mAb #4904

Rabbit monoclonal antibody to Phospho-p44/42 MAPK
(Erk1/2) (Thr202/Tyr204) (D13.14.4E) CST Cat #4370

Rabbit monoclonal antibody to p44/42 MAPK
(Erk1/2) (137F5) CST Cat #4695

Mouse monoclonal antibody to alpha-Tubulin
(clone B-5-1-2) Sigma Aldrich, Cat T6074

Full unedited gel for Figure 8G

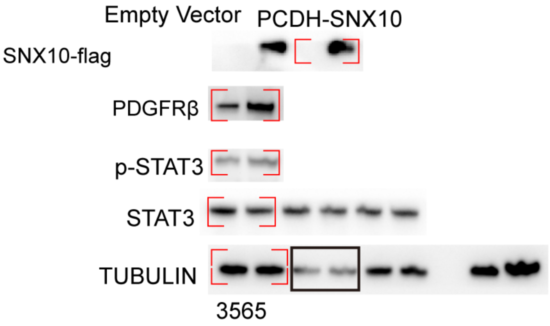

PDGF Receptor β (28E1) Rabbit mAb CST #3169

STAT3(79D7) Rabbit mAb #4904

Phospho-Stat3 (Tyr705) (D3A7) XP® Rabbit mAb CST#9145

Monoclonal ANTI-FLAG® M2 antibody produced in mouse (Clone M2) Sigma F1804

Mouse monoclonal antibody to alpha-Tubulin
(clone B-5-1-2) Sigma Aldrich, Cat T6074

Full unedited gel for Figure 10A

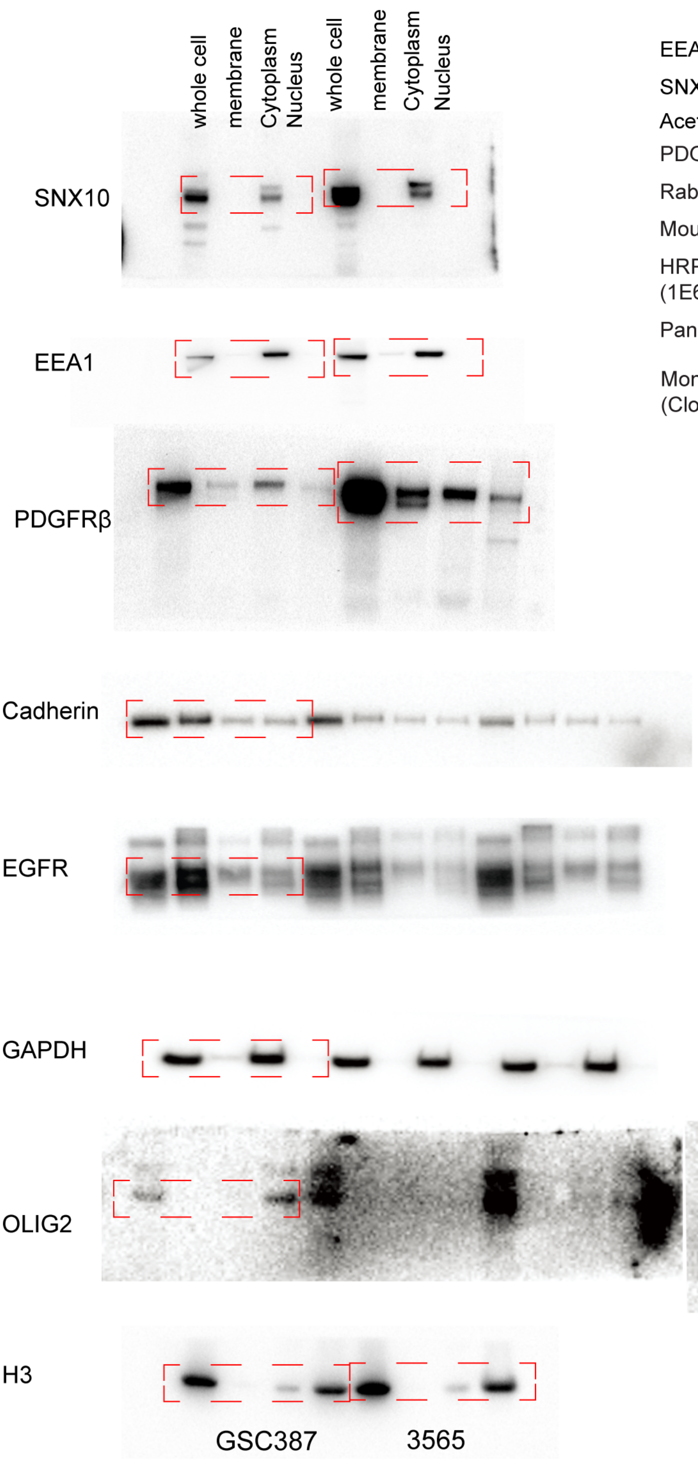

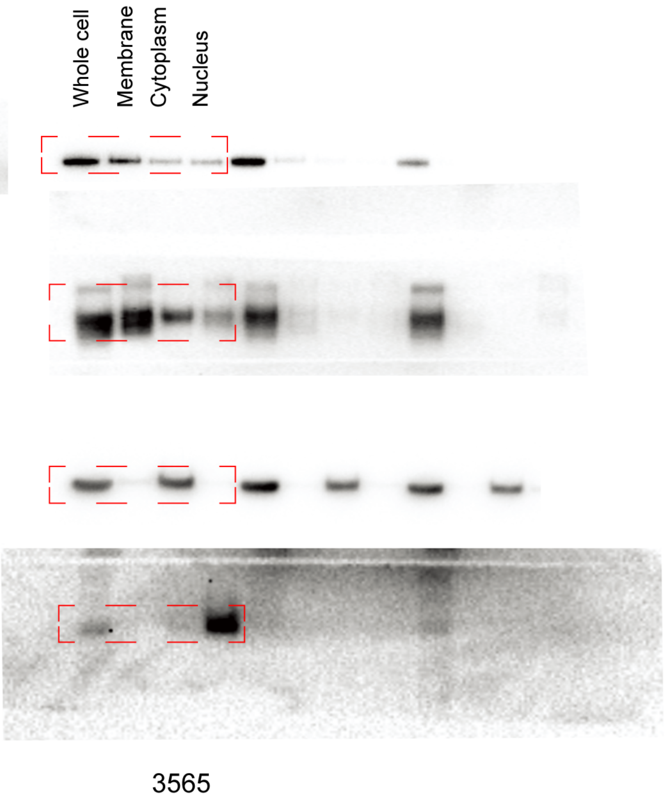

EEA1 (C45B10) Rabbit mAb CST #3288

SNX10 Monoclonal Antibody (OTI3F1) Origene Cat # TA808884

Acetyl-Histone H3 (Lys9) (C5B11) Rabbit mAb CST #9649

PDGF Receptor β (28E1) Rabbit mAb CST #3169

Rabbit monoclonal antibody to EGF Receptor (D38B1) CST Cat 4267

Mouse monoclonal antibody to OLIG2 (clone 211F1.1) EMD Millipore Cat MABN50

HRP-conjugated GAPDH Monoclonal antibody
(1E6D9) Proteintech Cat HRP-60004

Pan-Cadherin (28E12) Rabbit mAb #4073

Monoclonal ANTI-FLAG® M2 antibody produced in mouse
(Clone M2) Sigma F1804

Full unedited gel for Figure 10C

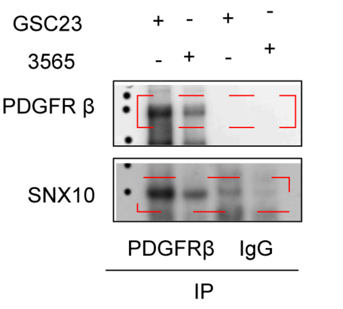

Full unedited gel for Figure 10D

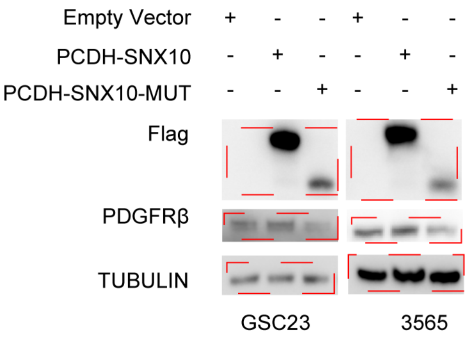

Full unedited gel for Figure 11C

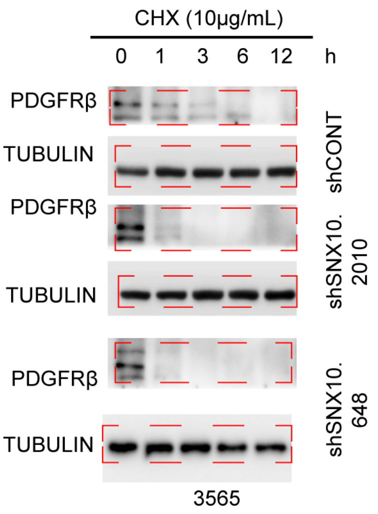

Full unedited gel for Figure 11E

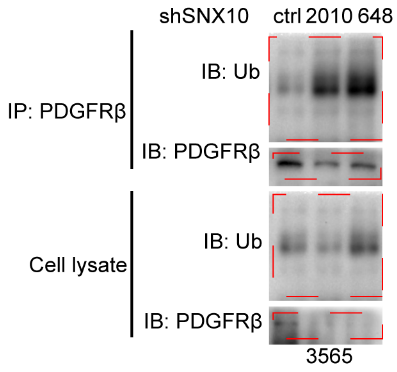

PDGF Receptor β (28E1) Rabbit mAb CST #3169

Mouse monoclonal antibody to alpha-Tubulin
(clone B-5-1-2) Sigma Aldrich, Cat T6074

Ubiquitin (E4I2J) Rabbit mAb CST #43124

Full unedited gel for Figure S3A

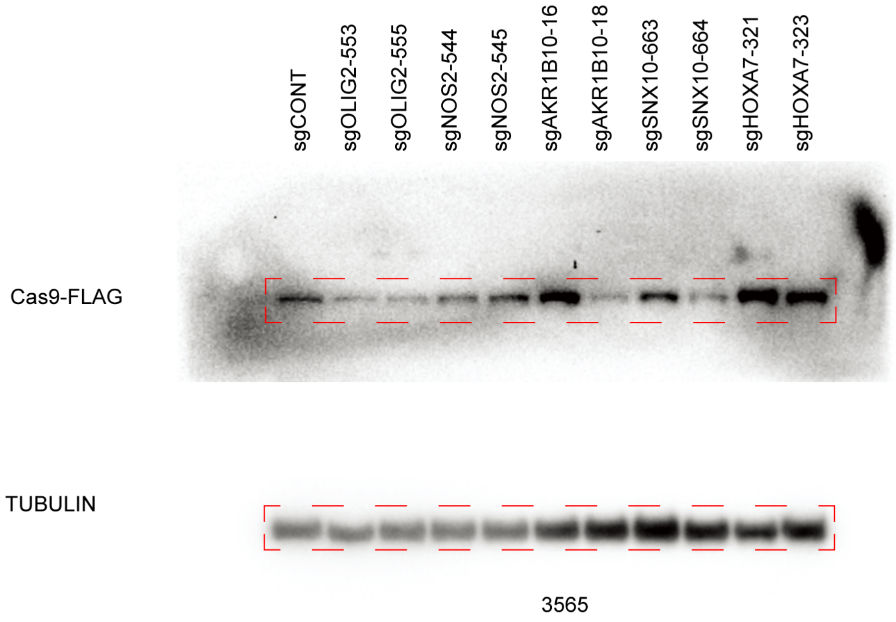

Monoclonal ANTI-FLAG® M2 antibody produced in mouse (Clone M2) Sigma F1804

Mouse monoclonal antibody to alpha-Tubulin
(clone B-5-1-2) Sigma Aldrich, Cat T6074

SNX10 Monoclonal Antibody (OTI3F1) Origene Cat # TA808884

Full unedited gel for Figure S4B-D

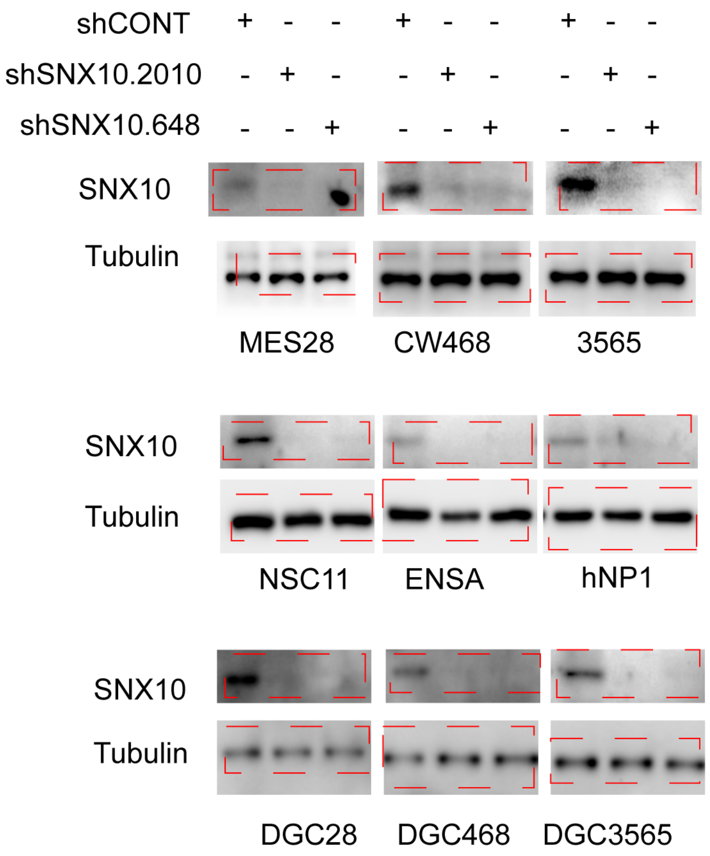

Full unedited gel for Figure S5C

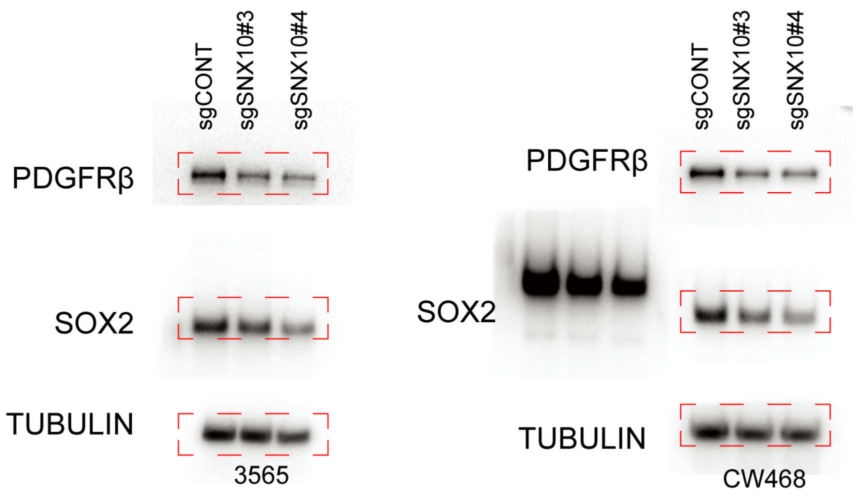

PDGF Receptor β (28E1) Rabbit mAb CST #3169

Goat polyclonal antibody to SOX2 R&D Systems Cat # AF2018

Mouse monoclonal antibody to alpha-Tubulin
(clone B-5-1-2) Sigma Aldrich, Cat T6074

Full unedited gel for Figure S5D

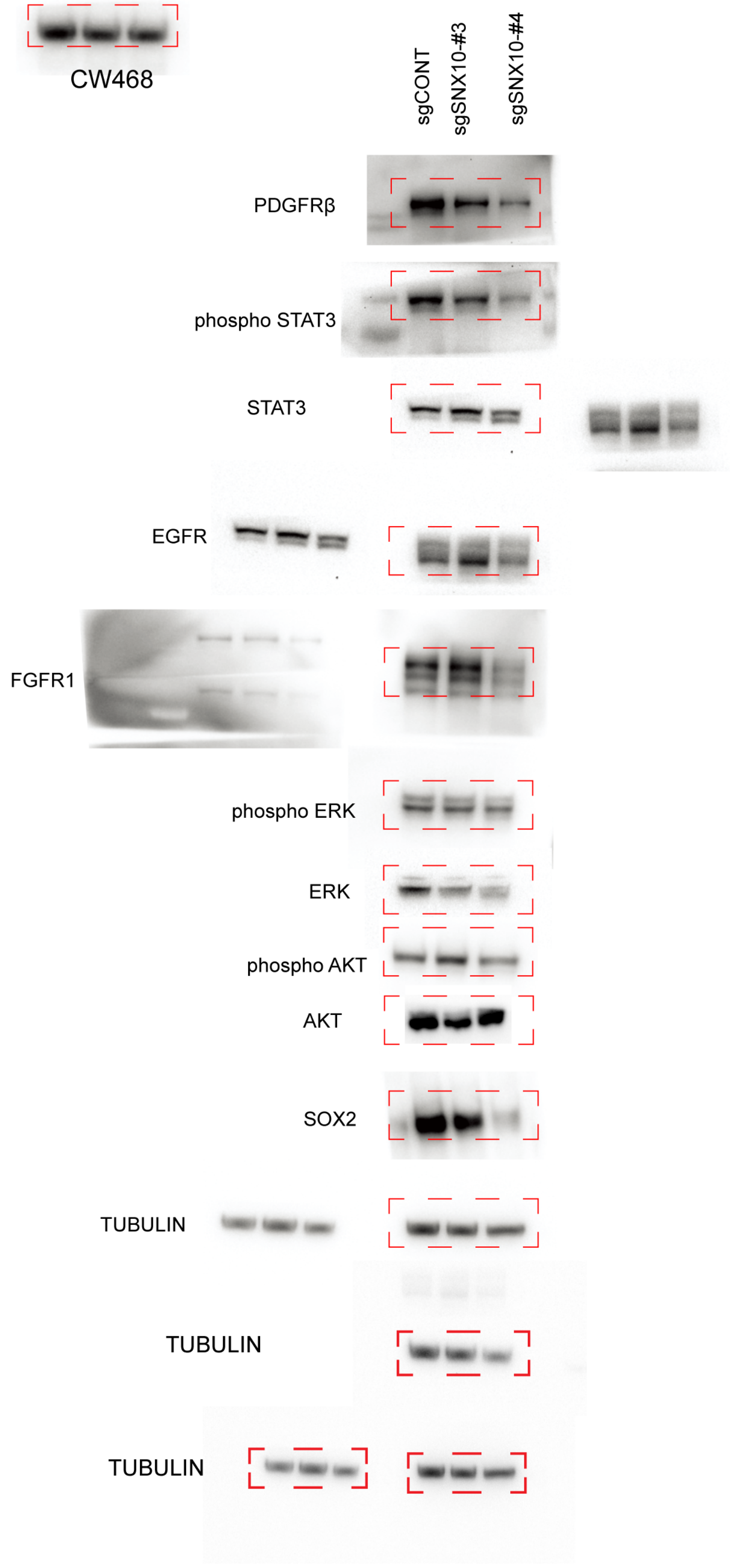

PDGF Receptor β (28E1) Rabbit mAb CST #3169

Phospho-Stat3 (Tyr705) (D3A7) XP® Rabbit mAb CST#9145

STAT3(79D7) Rabbit mAb #4904

Rabbit monoclonal antibody to EGF Receptor
(D38B1) CST Cat # 4267

FGF Receptor 1 (D8E4) XP® Rabbit mAb
#9740 CST Cat #9740

Rabbit monoclonal antibody to Phospho-p44/42 MAPK
(Erk1/2) (Thr202/Tyr204) (D13.14.4E) CST Cat #4370

Rabbit monoclonal antibody to p44/42 MAPK
(Erk1/2) (137F5) CST Cat #4695

Rabbit polyclonal antibody to Phospho-Akt
(Ser473) (D9E) CST Cat # 9271

Rabbit monoclonal antibody to AKT
(clone C67E7) CST Cat # 4691

Goat polyclonal antibody to SOX2 R&D Systems Cat # AF2018

Mouse monoclonal antibody to alpha-Tubulin
(clone B-5-1-2) Sigma Aldrich, Cat T6074

Full unedited gel for Figure S5E

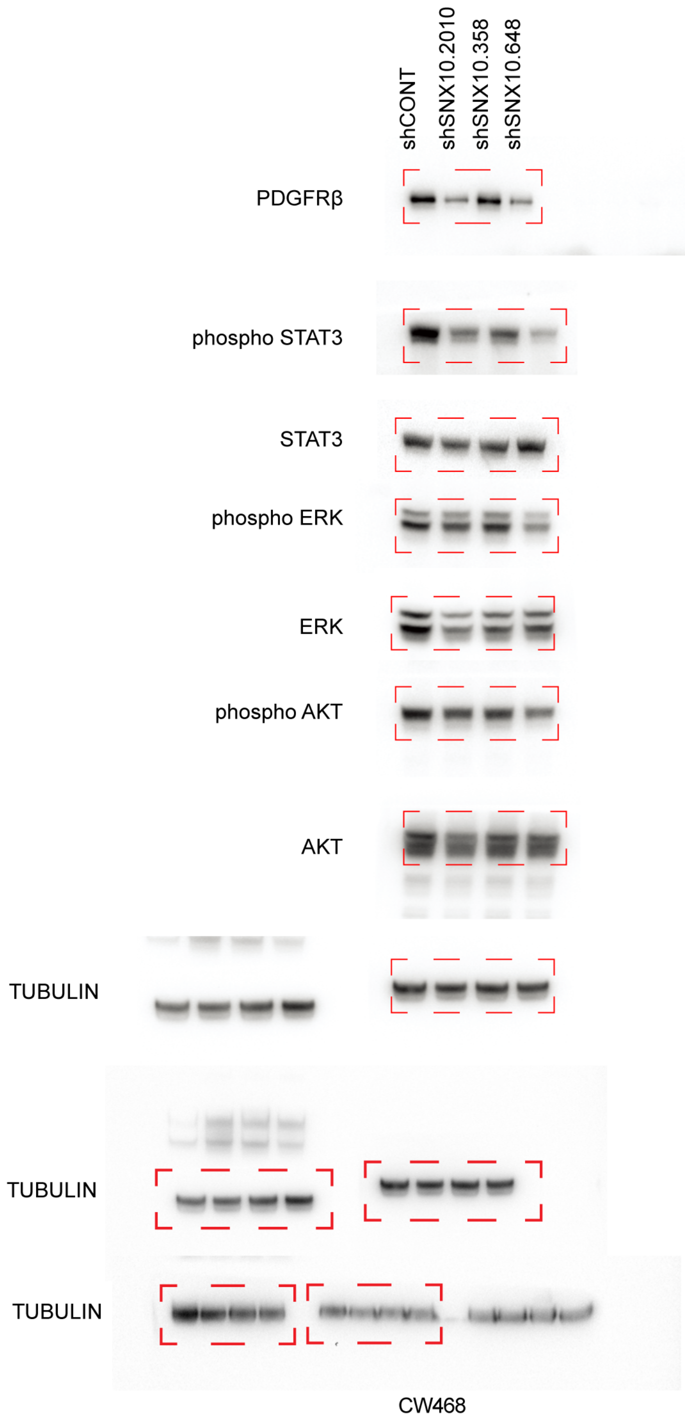

PDGF Receptor β (28E1) Rabbit mAb CST #3169

Phospho-Stat3 (Tyr705) (D3A7) XP® Rabbit mAb CST#9145

STAT3(79D7) Rabbit mAb #4904

Rabbit monoclonal antibody to Phospho-p44/42 MAPK (Erk1/2) (Thr202/Tyr204) (D13.14.4E) CST Cat #4370

Rabbit monoclonal antibody to p44/42 MAPK (Erk1/2) (137F5) CST Cat #4695

Rabbit polyclonal antibody to Phospho-Akt (Ser473) (D9E) CST Cat # 9271

Rabbit monoclonal antibody to AKT (clone C67E7) CST Cat # 4691

Mouse monoclonal antibody to alpha-Tubulin (clone B-5-1-2) Sigma Aldrich, Cat T6074

Ubiquitin (E4I2J) Rabbit mAb CST #43124

Monoclonal ANTI-FLAG® M2 antibody produced in mouse (Clone M2) Sigma F1804
